# Transcatheter aortic valve implantation vs. surgery for failed bioprosthesis: a meta-analysis of over 20 000 patients

**DOI:** 10.2459/JCM.0000000000001702

**Published:** 2025-01-20

**Authors:** Giuseppe Comentale, Armia Ahmadi-Hadad, Harvey James Moldon, Andreina Carbone, Rachele Manzo, Anna Franzone, Raffaele Piccolo, Eduardo Bossone, Giovanni Esposito, Emanuele Pilato

**Affiliations:** a Division of Cardiac Surgery; b Division of Cardiology, Department of Advanced Biomedical Sciences, University of Naples ‘Federico II’; c Division of Cardiology, University of Campania ‘L. Vanvitelli’; d Department of Public Health, University of Naples ‘Federico II’, Naples, Italy

**Keywords:** aortic regurgitation, aortic valve stenosis, bioprosthesis degeneration, redo surgical aortic valve replacement, valve-in-valve transcatheter aortic valve implantation

## Abstract

**Objectives:**

Valve-in-valve transcatheter aortic valve implantation (ViV-TAVI) has gained popularity as a less invasive alternative to a redo surgical aortic valve replacement (redo-SAVR); which one is the preferred technique in these cases, however, remains a topic of debate, as the available data refer to retrospective studies with few patients or limited follow-up. The present metanalysis aimed to compare the short-term and long-term outcomes of the two techniques in the setting of a failed surgical bioprosthesis.

**Methods:**

PubMed, MEDLINE, and Embase were searched on 10 November 2023 yielding 355 results (PROSPERO ID: CRD42023490612), of which 27 were suitable for meta-analysis. The primary outcomes were short-term and long-term all-causes and cardiovascular mortality. Logarithmic risk ratio (Log RR) and mean difference were used for categorical and continuous data, respectively.

**Results:**

Both redo-SAVR and ViV-TAVI exhibited similar procedural and short-term mortality. However, ViV-TAVI demonstrated lower 1-year mortality [RR: 0.74, 95% confidence interval (CI) (0.57–0.96), *P* = 0.02], acute kidney injury (RR: 0.47, *P* < 0.001), bleeding (RR: 0.44, *P* < 0.001), stroke (RR: 0.70, *P* < 0.05), and new pacemaker implantation (RR: 0.69, *P* < 0.05). Conversely, redo-SAVR demonstrated more favorable mean postoperative aortic valve gradients [mean difference 2.59, 95% CI (0.86–4.31), *P* < 0.01].

**Conclusion:**

Short-term mortality was similar between the groups, but ViV-TAVI showed better survival at 1 year as well as reduced rates of acute kidney injury, bleeding, stroke, and pacemaker implantation. However, redo-SAVR leads to a better hemodynamic profile. Even if collected data come from retrospective studies, the present results could help to guide the choice of the best approach case-by-case according to the patient's clinical profile.

## Graphical abstract

**Figure:**
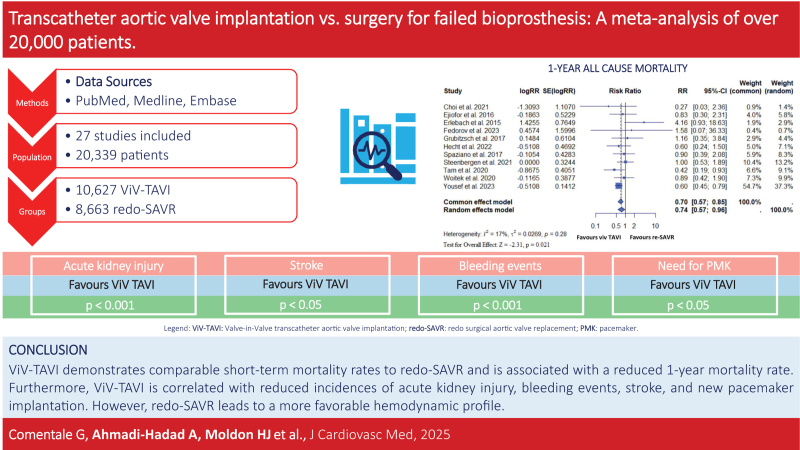

## Introduction

Aortic bioprosthetic valve degeneration is a common event after aortic valve replacement, and may require a surgical reintervention [redo surgical aortic valve replacement (redo-SAVR)] or a valve-in-valve transcatheter aortic valve implantation (ViV-TAVI), according to the preoperative risk profile.^1^ ViV-TAVI has gained popularity over the years as a less invasive method to treat aortic valve stenosis, as it does not require cardiopulmonary bypass (CPB), sternotomy, or cardioplegic arrest.^1–3^ For these reasons, ViV-TAVI could minimize the surgical risk, and, in the setting of redo-aortic valve surgery, it could help treat even complex patients at high risk for reoperation. Whether ViV-TAVI or redo-SAVR is the safer technique in these cases, and the advantages and disadvantages of each, remains a matter of debate. The available literature on the topic, indeed, mainly refers to observational or retrospective studies with a limited number of patients or with a short follow-up period,^4–30^ creating a significant gap in evidence that does not allow the derivation of strong conclusions. The limited data on long-term follow-up prevent a thorough discussion on this topic. The population with failing surgical bioprostheses typically consists of older and frailer patients, making the choice of treatment strategy crucial for their prognosis. This largely explains why a global clinical guideline for the use of ViV-TAVI or redo-SAVR for failed surgically implanted bioprosthetic valves has not yet been established. Many authors previously tried to summarize the available evidence by pooling data into metanalysis, suggesting that ViV-TAVI confers superior results in postoperative mortality but worse hemodynamic performances in the long term,^31^ but even highlighted the importance of keeping the data updated or designing focused controlled trials on the topic to provide stronger answers.

For these reasons, the present systematic review and meta-analysis aimed to update the existing evidence and explore the differences and the clinical outcomes of ViV-TAVI and redo-SAVR in failed surgical bioprosthetic valves.

## Materials and methods

**Fig. 1:**
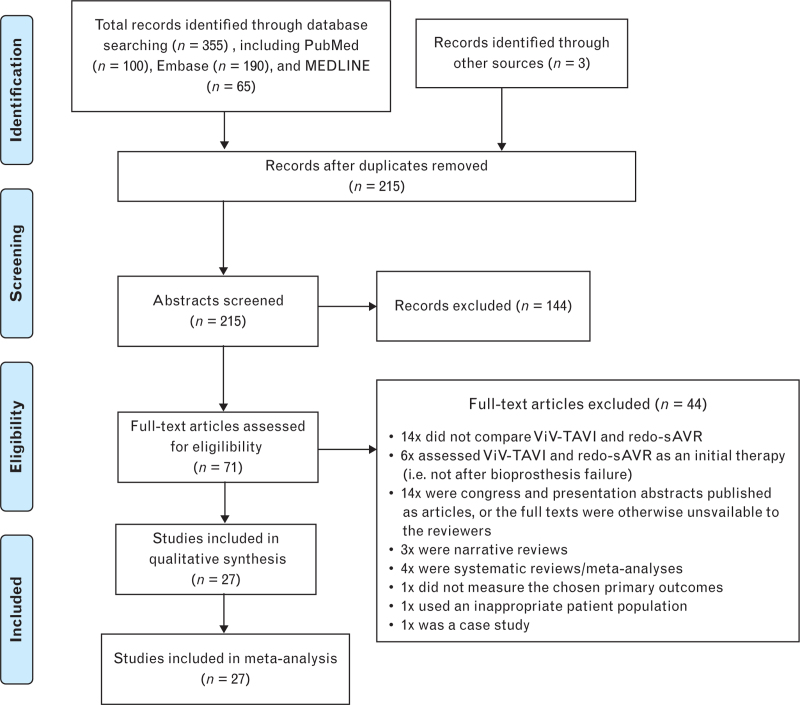
PRISMA flow chart demonstrating the screening and selection process.

PubMed, MEDLINE, and Embase were searched on 10 November 2023 yielding 215 results after the removal of duplicates. Exact search terms are available in the online supplementary data. Our protocol is registered with PROSPERO (CRD: CRD42023490612). Abstracts were screened and full texts were assessed for eligibility, during which bibliographies and citations were checked to identify additional studies. Eligible studies met the following criteria: retrospective studies must compare the use of ViV-TAVI to redo-SAVR following bioprosthetic valve failure; the study must provide data on at least one of four primary outcomes: procedural mortality, short-term mortality, 1-year mortality, cardiovascular mortality; and the full text was available to the reviewers. The screening and selection process including exclusions is described in Fig. 1. Our final selection included 27 studies that satisfied the criteria.^4–30^ All 27 studies were considered suitable for inclusion in the meta-analysis.^4–30^ The key characteristics of the included studies are summarized in Table 1.

**Table 1:** General overview of the studies included in the analysis

Year	Leading author	Use of propensity score matching	Patients receiving ViV-TAVI [*n* (%)]	Patients receiving redo-SAVR [*n* (%)]	Total patients	Country
2021	Choi *et al.*^4^	Unmatched	73 (42.2%)	100 (57.8%)	173	USA
2023	Cizmic *et al.*^5^	Unmatched	73 (81.12%)	17 (18.89%)	90	Germany
2012	Conradi *et al.*^6^	Matched	82 (50%)	82 (50%)	164	Germany
2020	Deharo *et al.*^7^	Matched	717 (50%)	717 (50%)	1434	France
2022	Demal *et al.*^8^	Unmatched	209 (76.28%)	65 (23.72%)	274	Germany
2021	Dokollari *et al.*^9^	Unmatched	31 (35.23%)	57 (64.77%)	88	Canada
2016	Ejiofor *et al.*^10^	Matched	22 (50%)	22 (50%)	44	USA
2015	Erlebach *et al.*^11^	Unmatched	50 (49.02%)	52 (50.98%)	102	Germany
2023	Fedorov *et al.*^12^	Unmatched	37 (66.07%)	19 (33.93%)	56	Germany
2023	Gatta *et al.*^13^	Matched	125 (50%)	125 (50%)	250	UK
2017	Grubitzsch *et al.*^14^	Unmatched	27 (51.92%)	25 (48.08%)	52	Germany
2022	Hecht *et al.*^15^	Unmatched	80 (43.48%)	104 (56.52%)	184	Canada
2019	Hernández-Vaquero *et al.*^16^	Matched	57 (50%)	57 (50%)	114	Spain
2020	Hirji *et al.*^29^	Unmatched	3443 (50.52%)	3372 (49.48%)	6815	USA
2021	Majmundar *et al.*^28^	Unmatched	3724 (55.02%)	3045 (44.98%)	6769	USA
2020	Malik *et al.*^17^	Matched	710 (50%)	710 (50%)	1420	USA
2021	Patel *et al.*^30^	Unmatched	187 (68.5%)	86 (31.5%)	273	USA
2016	Santarpino *et al.*^18^	Unmatched	6 (42.86%)	8 (57.14%)	14	Italy
2019	Sedeek *et al.*^19^	Unmatched	90 (25.71%)	260 (74.29%)	350	USA
2017	Silaschi *et al.*^20^	Unmatched	71 (54.62%)	59 (45.38%)	130	UK and Germany
2017	Spaziano *et al.*^21^	Unmatched	79 (38.54%)	126 (61.46%)	205	Multiple
2020	Stankowski *et al.*^22^	Matched	68 (62.96%)	40 (37.04%)	108	Germany
2020	Tam *et al.*^23^	Matched	131 (50%)	131 (50%)	262	Canada
2021	van Steenbergen *et al.*^24^	Matched	165 (50%)	165 (50%)	330	Netherlands
2021	Vukadinovikj *et al.*^25^	Unmatched	25 (71.43%)	10 (28.57%)	35	Germany
2020	Woitek *et al.*^26^	Unmatched	147 (56.98%)	111 (43.02%)	258	Germany
2023	Yousef *et al.*^27^	Unmatched	198 (57.39%)	147 (42.61%)	345	USA

### Data extraction

Data were extracted by two investigators in duplicate and using piloted forms. Our primary outcomes were procedural mortality; short-term mortality (defined as death within 30 days after the procedure); 1-year mortality (defined as death reported at follow-up 1 year after the procedure); and cardiovascular mortality. Procedural, in-hospital, and 30-day mortality data were pooled for quantitative analysis of 30-day mortality. Length of follow-up for each study is reported in detail in Supplementary Table 1.

Our secondary clinical outcomes included adverse events occurring within 30 days of the procedure, including stroke or transient ischemic attack (TIA), myocardial infarction (MI), acute kidney injury (AKI), new pacemaker implantation, and subsequent bleeding events. In-hospital and 30-day adverse events were pooled and analyzed together. Definitions of adverse events used by each study are reported in Supplementary Table 2. Additionally, echocardiographic postoperative aortic valve mean pressure gradient (AVG) and the number of patients with postoperative AVG greater than 20 mmHg were included as secondary outcomes.

Data included demographic and clinical characteristics of each cohort, such as age, female sex, BMI, diabetes, hypertension, dyslipidemia, tobacco use, chronic obstructive pulmonary disease (COPD), and coronary artery disease (CAD). Preoperative and postoperative echocardiographic data were collected, namely preoperative left ventricular ejection fraction (preop LVEF), postoperative left ventricular ejection fraction (postop LVEF), and preoperative AVG (preop AVG). Peri-operative data were extracted, including aortic cross-clamp time, time spent on CPB, and operation duration. These data are summarized in Supplementary Tables 3–7.

When studies employed propensity score matching, both matched and unmatched data were provided. In such cases, the matched data were used for analysis. In the case of Stankowski *et al.*,^22^ matched data were stratified by risk and presented as mean ± SD, and therefore could not be pooled. Only in this case were unmatched data used in place of available matched data.

### Statistics

Statistical analyses were conducted using R Studio (version 4.4.1), Revman (version 5.1), and OpenMEE.^32^ Data are presented as mean ± standard deviation or as median (interquartile range, IQR) for continuous variables, according to how they were reported in primary studies, and frequency (percentage) for categorical variables. The outcomes were estimated using the risk ratio (RR) with 95% confidence interval (CI) for all variables. Random effects models were used based on the DerSimonian–Laird method for analyses because it allowed a more conservative assessment of the pooled effect size. *I*^2^ was calculated as a measure of variation across studies caused by heterogeneity rather than chance. *I*^2^-values were interpreted as follows: less than 25%, 25–75%, and greater than 75%, representing low, medium, and high heterogeneity, respectively.^33^ Sources of potential heterogeneity were explored via sensitivity analysis, for which studies were removed one by one from each analysis to identify individual studies that may confer notable heterogeneity to the meta-analysis.

The RRs were initially calculated using RevMan. Subsequently, the forest plots were generated utilizing the metagen function within the R package ‘meta’ in RStudio. We have employed the logarithmic transformation of RR (Log RR) to stabilize the variance and address potential skewness in the distribution of effect sizes. However, in the case of procedural mortality, the logarithmic transformation did not result in model convergence. As a result, we analyzed the RR without logarithmic transformation for this specific outcome.

Meta-regressions were also conducted using a random-effects model. These were carried out for all primary and secondary outcomes using 22 covariates, where sufficient data were available. At least three studies were required to report covariate data per outcome subgroup to carry out the regression. The full list of covariates is provided in the supplementary information (online supplementary data). Publication bias was qualitatively examined using funnel plots, representing intervention effect (in this case, RR or mean difference) against standard error of the effect. Egger's test was used to increase the power of the findings (Supplementary information 4). Risk of bias in nonrandomized studies was assessed using the ROBINS-I tool.^34^

## Results

The meta-analysis included a total of 20 339 patients, of whom 10 627 underwent ViV-TAVI and 8663 underwent redo-SAVR. ViV-TAVI patients were significantly older at the time of procedure than redo-SAVR patients (13/27 studies), with higher rate of women in the ViV-TAVI group (in 2/27 studies). EuroSCORE II and the rate of III/IV NYHA class were significantly higher in the ViV-TAVI group in 6/27 studies and 3/27 studies, respectively (summarized in Supplementary Tables 4 and 7).

Procedural mortality was similar in both ViV-TAVI and redo-SAVR [RR: 0.62, 95% CI (0.31–1.24), *P* = 0.18, *I*^2^ = 23%] (Fig. 2 a). In the same vein, 30-day mortality was comparable between ViV-TAVI and redo-SAVR [RR: 0.72, 95% CI (0.49–1.04), *P* = 0.08, *I*^2^ = 48.6%] (Fig. 2 b). However, ViV-TAVI was associated with a significantly lower risk of 1-year mortality [RR: 0.74, 95% CI (0.57–0.96), *P* = 0.02, *I*^2^ = 17%] (Fig. 2 c). Moreover, cardiovascular mortality was evaluated in a small subset of studies reporting this outcome. Thirty-day cardiovascular mortality exhibited no significant difference between ViV-TAVI and redo-SAVR [RR: 0.57, 95% CI (0.32–1.01), *P* = 0.056, *I*^2^ = 0%] (Fig. 3a). Similarly, 1-year cardiovascular mortality was nonsignificant [RR: 1.06, 95% CI (0.52–2.17), *P* = 0.865, *I*^2^ = 0%] (Fig. 3b).

**Fig. 2:**
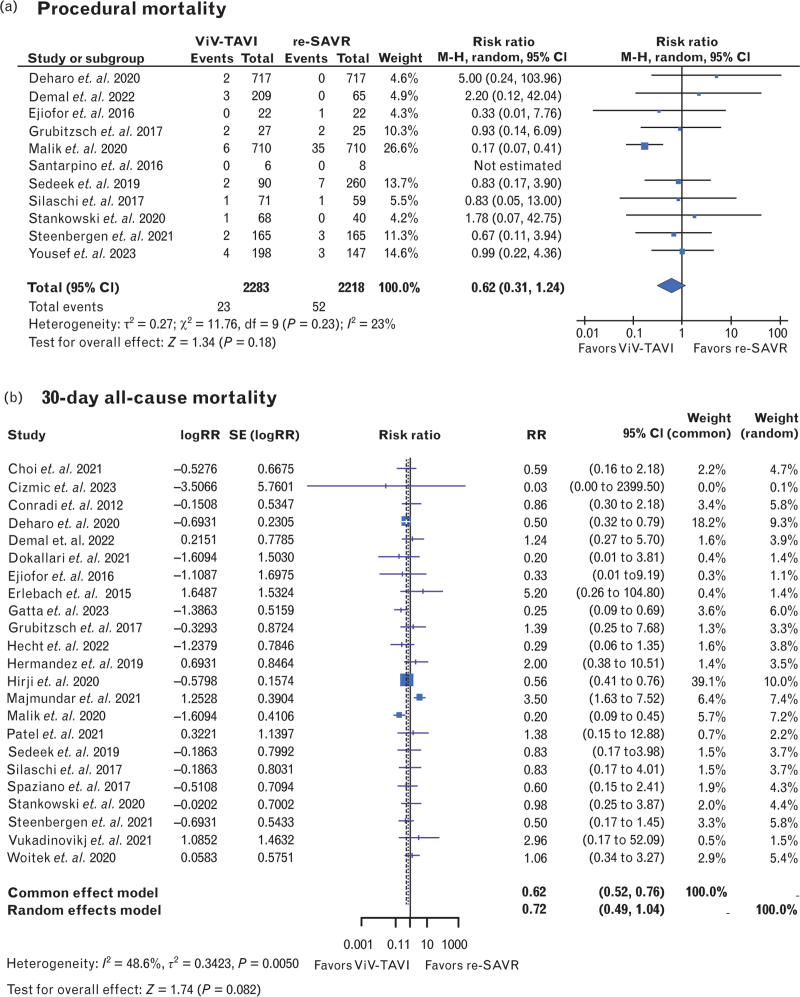
Forest plots comparing repeat valve-in-valve transcatheter aortic valve insertion and repeat surgical aortic valve replacement in terms of (a) procedural mortality; (b) 30-day all-cause mortality; (c) 1-year all-cause mortality. Redo-SAVR, repeat surgical aortic valve replacement; ViV-TAVI, valve-in-valve transcatheter aortic valve insertion.

**Fig. 2 (Continued):**
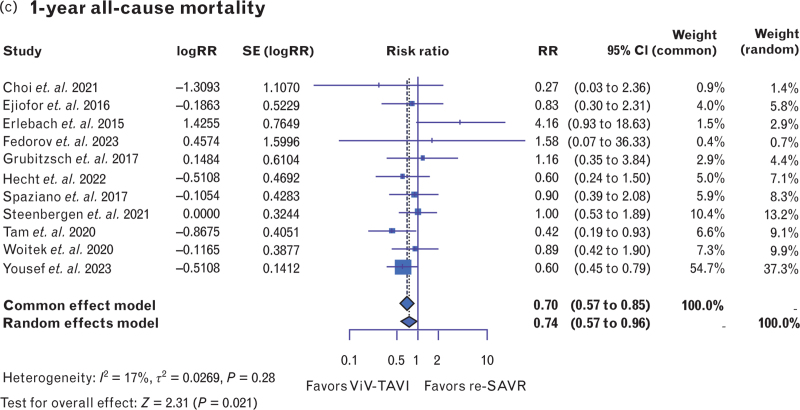
Forest plots comparing repeat valve-in-valve transcatheter aortic valve insertion and repeat surgical aortic valve replacement in terms of (a) procedural mortality; (b) 30-day all-cause mortality; (c) 1-year all-cause mortality. Redo-SAVR, repeat surgical aortic valve replacement; ViV-TAVI, valve-in-valve transcatheter aortic valve insertion.

**Fig. 3:**
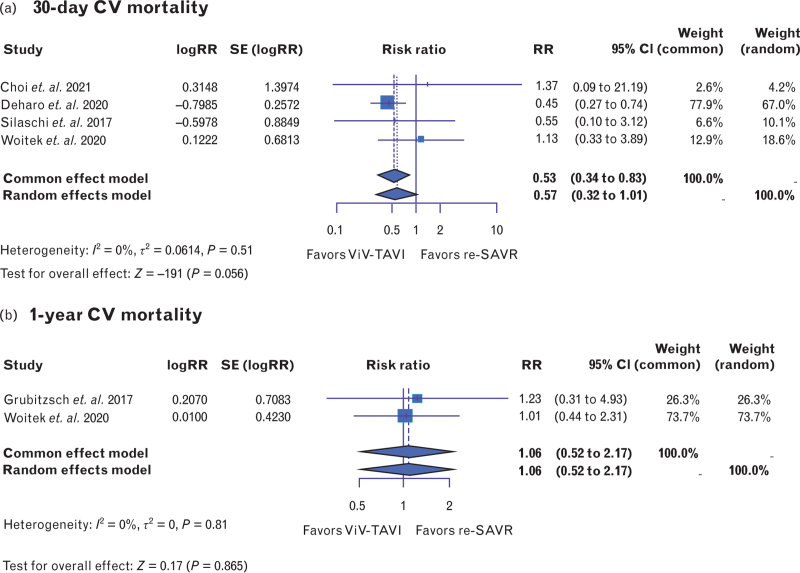
Forest plots comparing repeat valve-in-valve transcatheter aortic valve insertion and repeat surgical aortic valve replacement in terms of (a) 30-day mortality due to cardiovascular causes and (b) 1-year mortality due to cardiovascular causes. Redo-SAVR, repeat surgical aortic valve replacement; ViV-TAVI, valve-in-valve transcatheter aortic valve insertion.

In terms of short-term adverse events, ViV-TAVI was associated with a significantly lower risk of AKI [RR: 0.47, 95% CI (0.32–0.70), *P* < 0.001, *I*^2^ = 61.5%] (Fig. 4 a). Sensitivity analysis revealed that Hirji *et al.*^29^ contributed 26% to the heterogeneity of this analysis. Bleeding events proved to be significantly reduced in the ViV-TAVI group [RR: 0.44, 95% CI (0.31–0.62), *P* < 0.001, *I*^2^ = 93.8%] (Fig. 4 b). Sensitivity analysis demonstrated that Hirji *et al.*^29^ contributed 26% to the heterogeneity of this analysis. Similarly, rates of new heart block requiring pacemaker implantation were significantly lower in the ViV-TAVI group [RR: 0.69, 95% CI (0.48–0.99), *P* < 0.05, *I*^2^ = 83.4%] (Fig. 4 c).

**Fig. 4:**
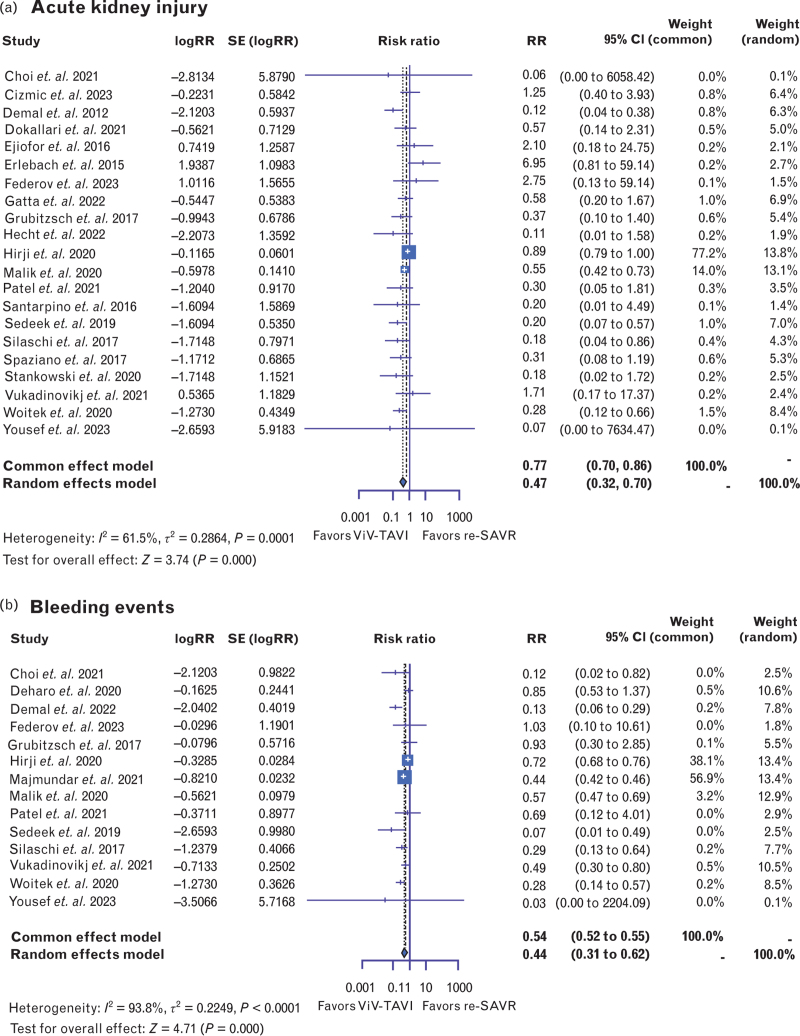
Forest plots comparing (a) acute kidney injury; (b) bleeding events; and (c) new pacemaker implantation within 30 days of new valve-in-valve transcatheter aortic valve insertion and repeat surgical aortic valve replacement. AKI, acute kidney injury; Redo-SAVR, repeat surgical aortic valve replacement; ViV-TAVI, valve-in-valve transcatheter aortic valve insertion.

**Fig. 4 (Continued):**
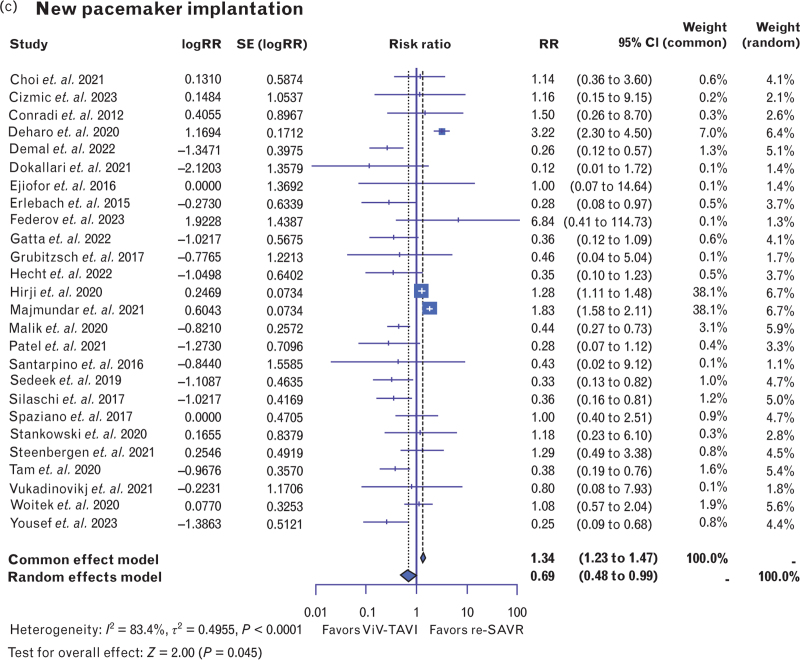
Forest plots comparing (a) acute kidney injury; (b) bleeding events; and (c) new pacemaker implantation within 30 days of new valve-in-valve transcatheter aortic valve insertion and repeat surgical aortic valve replacement. AKI, acute kidney injury; Redo-SAVR, repeat surgical aortic valve replacement; ViV-TAVI, valve-in-valve transcatheter aortic valve insertion.

**Fig. 5:**
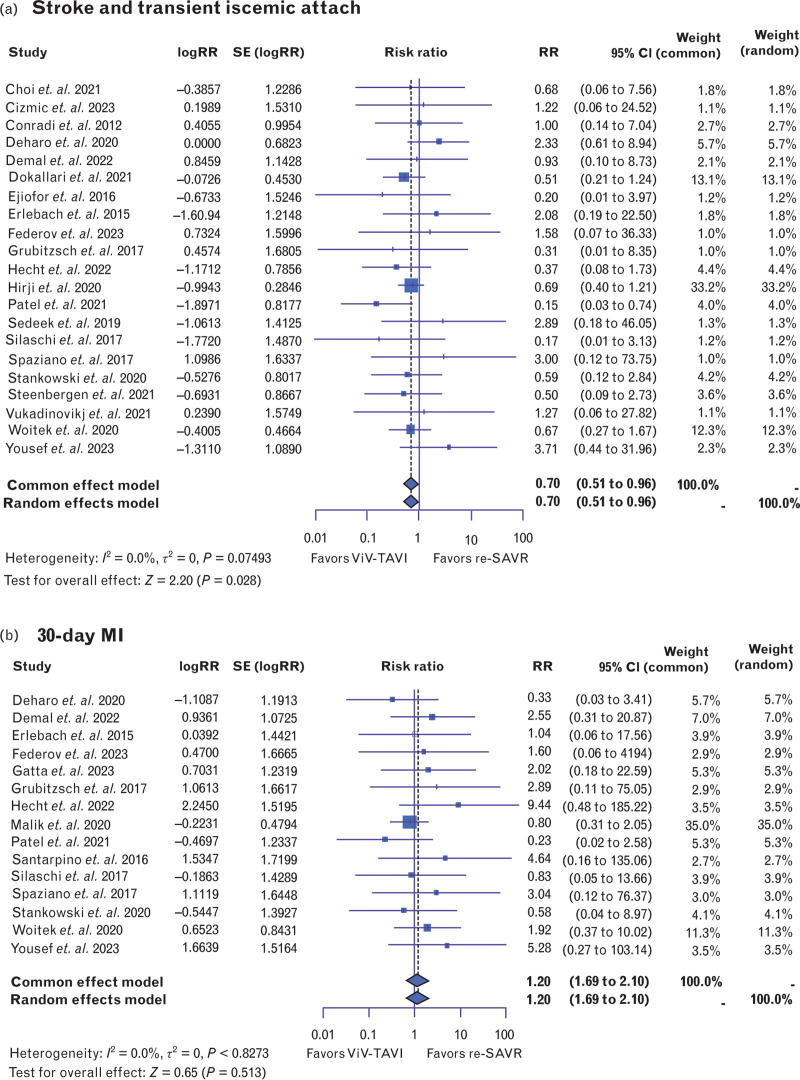
Forest plots comparing risk ratios of (a) stroke or transient ischemic attack; (b) myocardial infarction occurring within 30 days of valve-in-valve transcatheter aortic valve insertion or redo surgical aortic valve replacement. Redo-SAVR, repeat surgical aortic valve replacement; ViV-TAVI, valve-in-valve transcatheter aortic valve insertion.

Moreover, short-term risk of stroke or TIA was lower in ViV-TAVI compared with the redo-SAVR procedure [RR: 0.70, 95% CI (0.51–0.96), *P* < 0.05, *I*^2^ = 0%] (Fig. 5a); however, short-term risk of MI was not significantly different between these two groups [RR: 1.20, 95% CI (0.69–2.10), *P* = 0.513, *I*^2^ = 0%] (Fig. 5b).

**Fig. 6:**
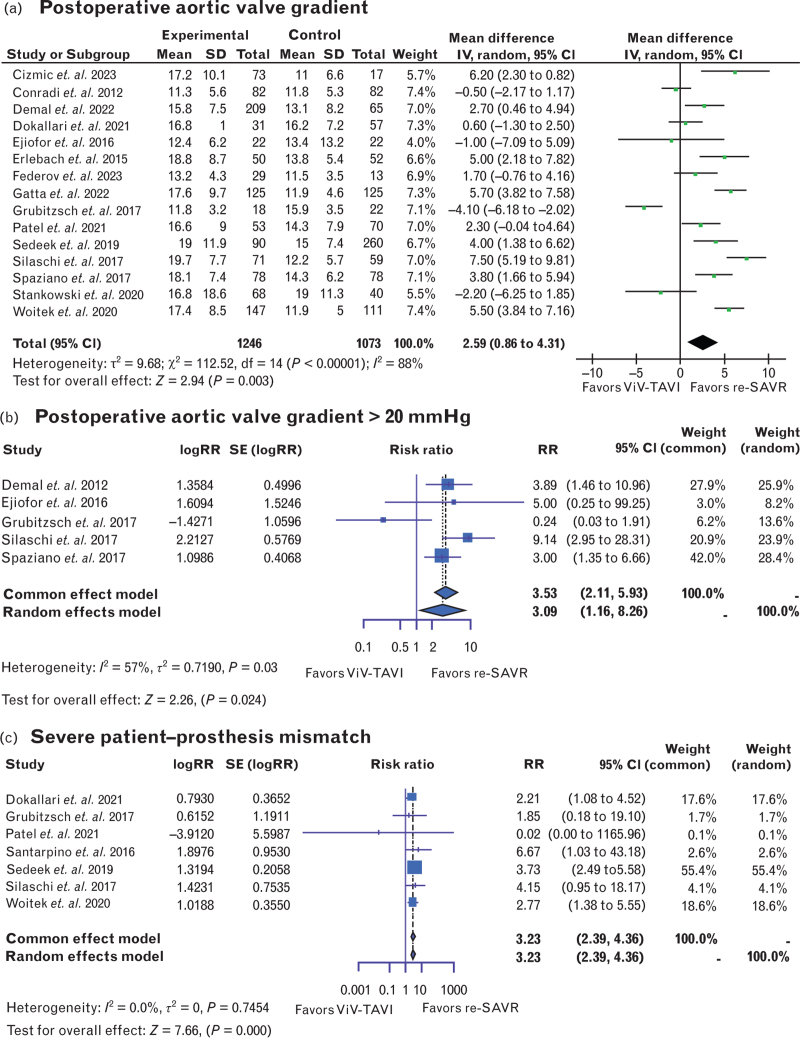
Forest plots comparing risk ratios of (a) mean postoperative aortic valve gradient; (b) the number of patients presenting with a postoperative aortic valve gradient greater than 20 mmHg; and (c) severe patient–prosthesis mismatch after valve-in-valve transcatheter aortic valve insertion or redo surgical aortic valve replacement. Redo-SAVR, repeat surgical aortic valve replacement; ViV-TAVI, valve-in-valve transcatheter aortic valve insertion.

In terms of echocardiographic parameters, postoperative AVG appears to be higher in ViV-TAVI patients [mean difference: 2.59, 95% CI (0.86–4.31), *P* < 0.01, *I*^2^ = 88%] (Fig. 6a). Additionally, the number of patients with a postoperative AVG greater than 20 mmHg was significantly higher in ViV-TAVI patients [RR: 3.09, 95% CI (1.16–8.26), *P* < 0.05, *I*^2^ = 57%] (Fig. 6b). Sensitivity analysis reveals that Grubitzsch *et al.*^14^ contributed for heterogeneity in this analysis, and its removal from the analysis reduced *I*^2^ to 0%. Patient–prosthesis mismatch was significantly more likely to occur in patients undergoing ViV-TAVI [RR: 3.23, 95% CI (2.39–4.36), *P* < 0.001, *I*^2^ = 0%] (Fig. 6c).

### Meta-regressions of primary and secondary outcomes

Meta-regression was carried out in all subgroups where at least three studies provided data for a given risk factor. Meta-regression of primary and secondary outcomes revealed various associations. Some of the most significant associations were between 1-year mortality and diabetes in ViV-TAVI (beta coefficient: −0.035, *P* = 0.04); between 1-year mortality and diabetes in redo-SAVR (beta coefficient: −0.039, *P* = 0.01); between 30-day mortality and peripheral artery disease in redo-SAVR (beta coefficient: −0.091, *P* = 0.002); between 30-day mortality and prior MI in redo-SAVR (beta coefficient: −0.101, *P* = 0.001); between new pacemaker implantation and prior CABG in ViV-TAVI (beta coefficient: −0.059, *P* = 0.001); between mean postop aortic valve gradient and hypertension in redo-SAVR (beta coefficient: −0.182, *P* = 0.002); and between mean postop aortic valve gradient and diabetes in redo-SAVR (beta coefficient: −0.206, *P* < 0.001). A complete list of associations is included in the online supplementary data.

Extending the analysis to include subgroup analysis was not feasible, as many of the included studies failed to report preoperative patient characteristics comprehensively or in a homogeneous manner.

### Risk of bias

Funnel plots appeared symmetrical, indicating that publication bias is unlikely to have affected the selected studies (Supplementary Figures 1–4). The ROBINS-I tool indicated only a moderate level of bias in most studies (Supplementary Figure 5).

## Discussion

Compared with redo-SAVR, ViV-TAVI is being increasingly recognized as a less invasive treatment for failing bioprosthetic valves. Many studies are currently lowering the cut-off age for surgical bioprosthetic valve implantation owing to the availability of longer-term data on the durability and safety of transcatheter valves, intending to save patients from lifelong anticoagulant therapy without exposing them to the risks associated with an early redo-cardiac surgery.^35^

This meta-analysis demonstrated a significantly lower 1-year all-cause mortality rate for patients receiving ViV-TAVI compared with those receiving redo-SAVR. Procedural mortality and 30-day mortality are similar between the two groups. However, redo-SAVR appeared to ensure the best results in terms of hemodynamic performance, as it allows lower postop-AVG, with a significantly reduced risk of patient–prosthesis mismatch. In the context of a patient-based approach, ViV-TAVI, therefore, represents a potentially preferable solution for older patients, for whom the risk of postoperative complications associated with a more invasive surgical procedure would outweigh the long-term benefits of a lower AVG.^36,37^ However, redo-SAVR is often carried out with concomitant CABG, which could increase the survival of younger patients.

The age of patients is probably one of the main issues to focus on and to consider when deciding on the treatment plan in the presence of a failing bioprosthesis; in this view, the data on follow-up probably represent the main determinant of the final choice. Gatta *et al.*^13^ and Hecht *et al.*^15^ both reported the longest follow-up on the topic, but unfortunately, they have conflicting results.

A recent study by Sá *et al.*^38^ attempted to provide a comprehensive overview by reconstructing time-to-event data up to 8 years. Their findings indicated that the hazard ratio is not constant over time, with ViV-TAVI producing more favorable outcomes in the initial 0.5 years, beyond which redo-SAVR is favored. Our analysis, which demonstrated better AVG results for the redo-SAVR group (Fig. 6a and b), holds the potential to explain these findings. However, it is important to note that, their study reported restricted mean survival time (RMST) for both ViV-TAVI and redo-SAVR exceeding 5 years (5.7 and 6.3 years, respectively), which surpasses the common follow-up duration of 5 years for the included studies. This suggested that the results may be disproportionately influenced by the two above-mentioned outlier studies with longer follow-up durations (Gatta *et al.*^13^ and Hecht *et al.*,^15^ which report 6-year and 8-year follow-ups, respectively). There is, therefore, a critical need for randomized controlled trials and primary studies with extended follow-up periods to further explore and clarify these findings.

Overall, echocardiographic data on postoperative AVG were less available, less complete, and more heterogenous than other data types. The high heterogeneity in these analyses (Fig. 6a and b) is likely because of greater variation in sample sizes arising from the inconsistent availability of echocardiographic data across studies. For example, Erlebach *et al.*^11^ reported that 24% of ViV-TAVI patients presented with a postoperative mean AVG greater than 25 mmHg, while redo-SAVR data were not provided and described as ‘incomplete’. However, the present results align with those observed in most available literature, which report better hemodynamic outcomes and significantly lower AVG among patients who have undergone redo-SAVR. This could explain why redo-SAVR patients experienced better long-term survival^38^ compared with ViV-TAVI but is surely something to consider when deciding the appropriate strategy for younger patients; definitely, lower mean AVG and lower probability of PPM are both prognosis-conditioning factors^36,37^ that could determine durability of the valve and the long-term mortality. In addition, this evidence should be seriously considered when treating patients with a small aortic annulus. These patients may benefit from surgical replacement as it can reduce the risk of PPM and physically enlarge the aortic annulus using techniques like the Nick, Y, or Manouguian procedures. This enlargement can facilitate a future ViV-TAVI with a larger prosthesis, thereby enhancing the valve's longevity.

It is noteworthy that our analysis is the first to yield significant findings for bleeding events, stroke, and pacemaker implantation, following the most comprehensive prior meta-analysis by Arjomandi Rad *et al.* (2022),^39^ which, in contrast, reported no superiority of either procedure with respect to these outcomes. The reduced risk of bleeding events makes ViV-TAVI an appealing option for patients with a history of bleeding disorders. On the other hand, redo-SAVR is often more appropriate for patients with functional renal comorbidities due to the use of a contrast medium in ViV-TAVI. In this analysis, however, ViV-TAVI demonstrated a significantly lower risk of 30-day AKI, which could be attributed to physicians’ appropriately selecting redo-SAVR in place of ViV-TAVI for patients with renal comorbidities.

Most studies suggested that ViV-TAVI appeared to be associated with a reduced risk of new pacemaker implantation after the intervention.^39,40^ On the other hand, Deharo *et al.*^7^ and Fedorov *et al.*^12^ both reported more pacemaker implantations in their ViV-TAVI cohorts. Fedorov *et al.*^12^ speculate that stented valves may offer greater protection for the atrial conduction system, leading to higher rates of pacemaker implantation in their research, which focused exclusively on degenerated stentless Freestyle valves. Deharo *et al.*^7^ unfortunately did not report data pertaining to bioprosthesis type. Overall, new pacemaker implantation risk was higher in redo-SAVR. Further investigation is warranted, particularly focusing on the types of bioprostheses used.

The occurrence and importance of systemic inflammatory response syndrome (SIRS) related to endovascular procedures in younger patients should also not be underestimated. Many authors have highlighted that endovascular interventions like ViV-TAVI can trigger SIRS leading to prolonged hospital stays, readmissions, major cardiovascular events, and reduced durability of the device.^41^ For all of the reasons outlined here, the choice between ViV-TAVI or redo-SAVR must be carefully evaluated and tailored to each specific patient.

### Limitations

As a meta-analysis of existing retrospective studies, these results were subject to disadvantages and biases of retrospective data. Although the ROBINS-I tool and funnel plots both indicated a low possibility for bias, a certain level of selection bias in retrospective studies is almost impossible to eliminate. The use of propensity-matched data helps to reduce this selection bias, but carrying out a meta-analysis exclusively on propensity-matched data is currently of limited scope. Moreover, it should be noted that the echocardiographic data on mean postoperative aortic valve gradients were less available, less complete, and more heterogenous than other types of data. The only technical limitation encountered was that in Fig. 2 a and Fig. 6a, the sole instances generated by RevMan; color modifications were not possible, thereby preventing alignment with the formatting of the remaining forest plots generated in R Studio.

## Conclusion

The present meta-analysis demonstrates that ViV-TAVI is associated with a lower 1-year mortality rate compared with redo-SAVR. Furthermore, ViV-TAVI is associated with lower AKI, bleeding events, pacemaker implantation, and stroke, which makes ViV-TAVI a more favorable option in elderly patients with comorbidities. However, redo-SAVR demonstrates superior hemodynamic outcomes, including postoperative aortic valve gradients and a reduced incidence of severe patient–prosthesis mismatch, which may have a potential impact on long-term survival beyond the 1-year mark.

Ultimately, the optimal option should be determined on a case-by-case basis, taking into account the clinical profile of the individual patient. Lastly, future investigations examining different bioprosthesis types are warranted to identify the optimal approach that minimizes the need for pacemaker implantation.

## Acknowledgements

Ethics/funding: As a meta-analysis, IRB approval and informed consent statements are not required.

Author contribution: G.C., A.A.-H. and H.J.M. share an equal contribution to the first author position. All the other authors equally contributed and accepted all parts of this manuscript.

Declaration of Generative AI and AI-assisted technologies in the writing process: During the preparation of this work, the authors used ChatGPT language model solely to improve readability. After using this tool, the authors reviewed and edited the content as needed and take full responsibility for the content of the publication.

### Conflicts of interest

There are no conflicts of interest.

## Supplementary Material

Supplemental Digital Content

## Supplementary Material

Supplemental Digital Content

## Supplementary Material

Supplemental Digital Content

## Supplementary Material

Supplemental Digital Content

## Supplementary Material

Supplemental Digital Content

## Supplementary Material

Supplemental Digital Content

## Supplementary Material

Supplemental Digital Content

## Data Availability

The data underlying this article are available in the article and in its online supplementary data.
