# Supplementary material for: Transcatheter aortic valve implantation vs. surgery for failed bioprosthesis: a meta-analysis of over 20 000 patients
Source: J Cardiovasc Med (Hagerstown). 2025 Jan 20;26(3):153–66. doi: 10.2459/JCM.0000000000001702 (PMC11841718; doi:10.2459/JCM.0000000000001702)
Supplement: Supplemental Digital Content [file jcarm-26-153-s001.docx]

**Supplementary Information 1: List of variables included in meta-regressions.**

**Variables included in the meta-regression across all outcomes:**

1. Mean age of patients undergoing TAVI
2. Mean age of patients undergoing sAVR
3. Percentage of female patients undergoing TAVI
4. Percentage of female patients undergoing sAVR
5. Percentage of diabetic patients undergoing TAVI
6. Percentage of diabetic patients undergoing sAVR
7. Percentage of patients with diagnosed hypertension undergoing TAVI
8. Percentage of patients with diagnosed hypertension undergoing sAVR
9. Mean pre-operative ejection fraction in patients undergoing TAVI
10. Mean pre-operative ejection fraction in patients undergoing sAVR
11. Percentage of patients with NYHA class III and class IV heart failure undergoing TAVI
12. Percentage of patients with NYHA class III and class IV heart failure undergoing sAVR
13. Percentage of patients with peripheral artery disease undergoing TAVI
14. Percentage of patients with peripheral artery disease undergoing sAVR
15. Percentage of patients with a history of atrial fibrillation undergoing TAVI
16. Percentage of patients with a history of atrial fibrillation undergoing sAVR
17. Percentage of patients with coronary artery disease undergoing TAVI
18. Percentage of patients with coronary artery disease undergoing sAVR
19. Percentage of patients with prior myocardial infarction undergoing TAVI
20. Percentage of patients with prior myocardial infarction undergoing sAVR
21. Percentage of patients with prior coronary artery bypass grafting undergoing TAVI
22. Percentage of patients with prior coronary artery bypass grafting undergoing sAVR

**Additional variables included in the meta-regression for 30-day stroke:**

1. Percentage of patients with prior stroke undergoing TAVI
2. Percentage of patients with prior stroke undergoing sAVR

**Additional variables included in the meta-regression for Acute kidney injury:**

1. Percentage of patients with chronic kidney disease undergoing TAVI
2. Percentage of patients with chronic kidney disease undergoing sAVR

**Additional variables included in the meta-regression for new pacemaker implantation:**

1. Percentage of patients with prior pacemaker undergoing TAVI
2. Percentage of patients with prior pacemaker undergoing sAVR

**Additional variables included in the meta-regression for severe patient prosthesis mismatch:**

1. Percentage of patients with prosthesis stenosis undergoing TAVI
2. Percentage of patients with prosthesis stenosis undergoing sAVR

**Additional variables included in the meta-regression for post-op aortic valve gradient:**

1. Percentage of patients with prosthesis regurgitation undergoing TAVI
2. Percentage of patients with prosthesis regurgitation undergoing sAVR
3. Percentage of patients with prosthesis stenosis undergoing TAVI
4. Percentage of patients with prosthesis stenosis undergoing sAVR
5. Mean aortic valve gradient in patients undergoing TAVI
6. Mean aortic valve gradient in patients undergoing sAVR

**Supplementary Information 2: List of associations identified for primary and secondary outcomes via meta-regression.**

No associations were found for the outcomes 30-day MI; 30-day stroke or TIA; or aortic valve gradient >20mmHg.

**1-year mortality**

Female sex in redo-SAVR (beta coefficient: +0.099, p=0.03)

Diabetes in ViV-TAVI (beta coefficient: -0.035, p=0.04)

Diabetes in redo-SAVR (beta coefficient: -0.039, p=0.01)

**30-day mortality**

Peripheral artery disease in redo-SAVR (beta coefficient: -0.091, p=0.002)

Prior MI in redo-SAVR (beta coefficient: -0.101, p=0.001)

**Procedural mortality**

Peripheral artery disease in redo-SAVR (beta coefficient: -0.173, p=0.01)

**Acute kidney injury**

Hypertension in ViV-TAVI (beta coefficient: -0.018, p<0.001)

Hypertension in redo-SAVR (beta coefficient: -0.027, p=0.001)

Prior CABG in ViV-TAVI (beta coefficient: -0.032, p=0.03)

**Bleeding events**

Prior MI in redo-SAVR (beta coefficient: +0.158, p=0.001)

Prior CABG in ViV-TAVI (beta coefficient: -0.045, p=0.04)

**Pacemaker**

Pre-op ejection fraction in ViV-TAVI (beta coefficient: +0.252, p=0.01)

Coronary artery disease in redo-SAVR (beta coefficient: +0.046, p=0.008)

Prior CABG in ViV-TAVI (beta coefficient: -0.059, p=0.001)

Prior Pacemaker in ViV-TAVI (beta coefficient: +0.079, p=0.04)

**Severe patient-prosthesis mismatch**

Age in ViV-TAVI (beta coefficient: +0.747, p=0.03)

**Post-op aortic valve gradient**

Diabetes in redo-SAVR (beta coefficient: -0.206, p<0.001)

Hypertension in redo-SAVR (beta coefficient: -0.182, p=0.002)

NYHA classification III/IV in redo-SAVR (beta coefficient: -0.119, p=0.004)

Atrial fibrillation in redo-SAVR (beta coefficient: -0.123, p=0.02)

Coronary artery disease in ViV-TAVI (beta coefficient: -0.102, p=0.03)

Coronary artery disease in redo-SAVR (beta coefficient: -0.194, p<0.001)

**Supplementary Information 3: Full search terms.**

**Pubmed:**

(“surgical redo”[tw] OR “surgical replacement”[tw] OR “redo surgery”[tw] OR “redo surgical AVR“[tw] OR “surgical rereplacement”[tw] OR “reoperative surgical aortic valve replacement”[tw] OR “Redo aortic Valve Replacement“[tw] OR “redo surgery”[tw] OR “aortic valve replacement“[tw] “redo surgical aortic valve replacement“[tw] OR “reoperative Surgical aortic Valve Replacement“[tw] OR “Redo procedures “[tw] OR “Redo aortic valve surgery”[tw] OR "redo surgical aortic valve implantation"[tw] OR "redo surgical aortic valve replacement"[tw] OR “AVR”[tw] OR “redo AVR”[tw] OR “re-sAVR”[tw] OR “re-AVR”[tw] OR “redo-SAVR”[tw] OR “surgery”[tw]) AND (“redo TAVR”[tw] OR “redo TAVI”[tw] OR “ViV TAVI”[tw] OR "transcatheter aortic valve-in-valve"[tw] OR "transcatheter aortic valve-in-valve replacement"[tw] OR "transcatheter aortic valve-in-valve implantation"[tw] OR “Valve in sAVR”[tw] OR “TAVI in SAVR”[tw] OR “TAVI in valve”[tw] OR “TAVI in AVR”[tw] OR “transcatheter redo AVR”[tw] OR “transcatheter re-AVR”[tw] OR ”transcatheter valve-in-valve”[tw] OR “valve-in-valve transcatheter”[tw] OR “Transcatheter aortic valve in-valve implantation”[tw] OR “Transcatheter aortic Valve-in-Valve implantation”[tw] OR “Valve-in-Valve Transcatheter”[tw] OR “Transcatheter aortic valve implantation”[tw] OR “valve‐in‐valve transcatheter aortic valve replacement”[tw] OR “Transcatheter ViV”[tw]) AND (“aort*”[tw] OR “SAVR”[tw] OR “TAVR”[tw] OR “TAVI”[tw] OR “AVR”[tw]) AND (“degenerated aort*”[tw] OR “redo”[tw] OR “Valve-in-Valve”[tw] OR “ViV”[tw] OR “reoperative”[tw] OR “re-AVR”[tw] OR “TAVI in SAVR”[tw] OR “TAVI in valve”[tw] OR “TAVI in AVR”[tw]) AND (“versus”[ti] OR “vs.”[ti] OR “vs”[ti] OR “strategies”[ti] OR “alternative”[ti] OR “evaluation”[ti] OR “compar*”[ti] OR “or”[ti] OR “meta”[ti] OR “meta-analysis”[ti]) NOT (“tricuspid”[ti] OR “mitral”[ti] OR “pulmonary”[ti] OR “balloon”[ti])

**Embase:**

('surgical redo':ti,ab,kw,de OR 'surgical replacement':ti,ab,kw,de OR 'redo surgery':ti,ab,kw,de OR 'redo surgical AVR':ti,ab,kw,de OR 'surgical rereplacement':ti,ab,kw,de OR 'reoperative surgical aortic valve replacement':ti,ab,kw,de OR 'Redo aortic Valve Replacement':ti,ab,kw,de OR 'redo surgery':ti,ab,kw,de OR 'aortic valve replacement':ti,ab,kw,de 'redo surgical aortic valve replacement':ti,ab,kw,de OR 'reoperative Surgical aortic Valve Replacement':ti,ab,kw,de OR 'Redo procedures':ti,ab,kw,de OR 'Redo aortic valve surgery':ti,ab,kw,de OR 'redo surgical aortic valve implantation':ti,ab,kw,de OR 'redo surgical aortic valve replacement':ti,ab,kw,de OR 'AVR':ti,ab,kw,de OR 'redo AVR':ti,ab,kw,de OR 're-sAVR':ti,ab,kw,de OR 're-AVR':ti,ab,kw,de OR 'redo-SAVR':ti,ab,kw,de OR 'surgery':ti,ab,kw,de ) AND ('redo TAVR':ti,ab,kw,de OR 'redo TAVI':ti,ab,kw,de OR 'ViV TAVI':ti,ab,kw,de OR 'transcatheter aortic valve-in-valve':ti,ab,kw,de OR 'transcatheter aortic valve-in-valve replacement':ti,ab,kw,de OR 'transcatheter aortic valve-in-valve implantation':ti,ab,kw,de OR 'Valve in sAVR':ti,ab,kw,de OR 'TAVI in SAVR':ti,ab,kw,de OR 'TAVI in valve':ti,ab,kw,de OR 'TAVI in AVR':ti,ab,kw,de OR 'transcatheter redo AVR':ti,ab,kw,de OR 'transcatheter re-AVR':ti,ab,kw,de OR 'transcatheter valve-in-valve':ti,ab,kw,de OR 'valve-in-valve transcatheter':ti,ab,kw,de OR 'Transcatheter aortic valve in-valve implantation':ti,ab,kw,de OR 'Transcatheter aortic Valve-in-Valve implantation':ti,ab,kw,de OR 'Valve-in-Valve Transcatheter':ti,ab,kw,de OR 'Transcatheter aortic valve implantation':ti,ab,kw,de OR 'valve‐in‐valve transcatheter aortic valve replacement':ti,ab,kw,de OR 'Transcatheter ViV':ti,ab,kw,de ) AND ('aort*':ti,ab,kw,de OR 'SAVR':ti,ab,kw,de OR 'TAVR':ti,ab,kw,de OR 'TAVI':ti,ab,kw,de OR 'AVR':ti,ab,kw,de ) AND ('degenerated aort*':ti,ab,kw,de OR 'redo':ti,ab,kw,de OR 'Valve-in-Valve':ti,ab,kw,de OR 'ViV':ti,ab,kw,de OR 'reoperative':ti,ab,kw,de OR 're-AVR':ti,ab,kw,de OR 'TAVI in SAVR':ti,ab,kw,de OR 'TAVI in valve':ti,ab,kw,de OR 'TAVI in AVR':ti,ab,kw,de ) AND ('versus':ti OR 'vs.':ti OR 'vs':ti OR 'strategies':ti OR 'alternative':ti OR 'evaluation':ti OR 'compar*':ti OR 'or':ti OR 'meta':ti OR 'meta-analysis':ti) NOT ('tricuspid':ti OR 'mitral':ti OR 'pulmonary':ti OR 'balloon':ti)

**Medline:**

(("surgical redo" OR "surgical replacement" OR "redo surgery" OR "redo surgical AVR" OR "surgical rereplacement" OR "reoperative surgical aortic valve replacement" OR "Redo aortic Valve Replacement" OR "redo surgery" OR "aortic valve replacement" "redo surgical aortic valve replacement" OR "reoperative Surgical aortic Valve Replacement" OR "Redo procedures " OR "Redo aortic valve surgery" OR "redo surgical aortic valve implantation" OR "redo surgical aortic valve replacement" OR "AVR" OR "redo AVR" OR "re-sAVR" OR "re-AVR" OR "redo-SAVR" OR "surgery") AND ("redo TAVR" OR "redo TAVI" OR "ViV TAVI" OR "transcatheter aortic valve-in-valve" OR "transcatheter aortic valve-in-valve replacement" OR "transcatheter aortic valve-in-valve implantation" OR "Valve in sAVR" OR "TAVI in SAVR" OR "TAVI in valve" OR "TAVI in AVR" OR "transcatheter redo AVR" OR "transcatheter re-AVR" OR "transcatheter valve-in-valve" OR "valve-in-valve transcatheter" OR "Transcatheter aortic valve in-valve implantation" OR "Transcatheter aortic Valve-in-Valve implantation" OR "Valve-in-Valve Transcatheter" OR "Transcatheter aortic valve implantation" OR "valve‐in‐valve transcatheter aortic valve replacement" OR "Transcatheter ViV") AND ("aort*" OR "SAVR" OR "TAVR" OR "TAVI" OR "AVR") AND ("degenerated aort*" OR "redo" OR "Valve-in-Valve" OR "ViV" OR "reoperative" OR "re-AVR" OR "TAVI in SAVR" OR "TAVI in valve" OR "TAVI in AVR")).tw. AND ("versus" OR "vs." OR "vs" OR "strategies" OR "alternative" OR "evaluation" OR "compar*" OR "or" OR "meta" OR "meta-analysis").ti. NOT ("tricuspid" OR "mitral" OR "pulmonary" OR "balloon").ti.

**Supplementary Information 4: Egger’s tests.**

1. **30-day Myocardial Infarction**

Test result: t = 1.57, df = 13, p-value = 0.1403; Bias estimate: 0.7662 (SE = 0.4878)

Details: multiplicative residual heterogeneity variance (tau^2 = 0.5856)

1. **30-day Mortality**

Test result: t = 1.02, df = 21, p-value = 0.3203 ; Bias estimate: 0.4813 (SE = 0.4729)

Details: multiplicative residual heterogeneity variance (tau^2 = 1.9412)

1. **Acute Kidney injuries**

Test result: t = -2.41, df = 19, p-value = 0.0262 ; Bias estimate: -0.8953 (SE = 0.3712)

Details: multiplicative residual heterogeneity variance (tau^2 = 2.0946)

1. **Bleeding events**

Test result: t = -0.36, df = 12, p-value = 0.7229 ; Bias estimate: -0.4719 (SE = 1.2996)

Details: multiplicative residual heterogeneity variance (tau^2 = 17.3545)

1. **New pacemaker implantation**

Test result: t = -3.25, df = 24, p-value = 0.0034 ; Bias estimate: -1.7271 (SE = 0.5312)

Details: multiplicative residual heterogeneity variance (tau^2 = 4.3496)

1. **Postop Aortic gradient greater than 20 mmHg**

Test result: t = -0.59, df = 3, p-value = 0.5944 ; Bias estimate: -1.1297 (SE = 1.9022)

Details: multiplicative residual heterogeneity variance (tau^2 = 2.8057)

1. **Severe patient prosthesis mismatch**

Test result: t = -0.86, df = 5, p-value = 0.4316 ; Bias estimate: -0.4196 (SE = 0.4908)

Details: multiplicative residual heterogeneity variance (tau^2 = 0.6088)

1. **Stroke**

Test result: t = 0.75, df = 19, p-value = 0.4645 ; Bias estimate: 0.2763 (SE = 0.3701)

Details: multiplicative residual heterogeneity variance (tau^2 = 0.7907)

1. **1-year mortality**

Test result: t = 1.50, df = 9, p-value = 0.1689 ; Bias estimate: 0.8175 (SE = 0.5465)

Details: multiplicative residual heterogeneity variance (tau^2 = 1.0716)

1. **30-day CV mortality**

Regression Test for Funnel Plot Asymmetry

Model: weighted regression with multiplicative dispersion

Predictor: standard error

Test for Funnel Plot Asymmetry: t = 1.6956, df = 2, p = 0.2321

Limit Estimate (as sei -> 0): b = -1.0314 (CI: -2.2369, 0.1740)

1. **Postop aortic valve gradients**

Regression Test for Funnel Plot Asymmetry

Model: weighted regression with multiplicative dispersion

Predictor: standard error

Test for Funnel Plot Asymmetry: t = -1.6210, df = 13, p = 0.1290

Limit Estimate (as sei -> 0): b = 0.9406 (CI: 0.2168, 1.6644)

1. **Procedural mortality**

Regression Test for Funnel Plot Asymmetry

Model: weighted regression with multiplicative dispersion

Predictor: standard error

Test for Funnel Plot Asymmetry: t = 3.3063, df = 9, p = 0.0091

Limit Estimate (as sei -> 0): b = -1.9673 (CI: -2.9890, -0.9455)
