# Supplementary material for: Transcatheter aortic valve implantation vs. surgery for failed bioprosthesis: a meta-analysis of over 20 000 patients
Source: J Cardiovasc Med (Hagerstown). 2025 Jan 20;26(3):153–66. doi: 10.2459/JCM.0000000000001702 (PMC11841718; doi:10.2459/JCM.0000000000001702)
Supplement: Supplemental Digital Content [file jcarm-26-153-s002.docx]

**Supplementary Table 1**

| Leading author | Year | Procedural mortality | Follow-ups for hort-term all-cause mortality | Follow-ups for mid-term / long-term all-cause mortality | Follow-ups for short term mortality due to cardiovascular causes | Follow-ups for mid-term / long-term mortality due to cardiovascular causes |
| --- | --- | --- | --- | --- | --- | --- |
| Choi | 2021 | *Outcome not investigated* | 30 days | 1 year | 30 days | 1 year |
| Cizmic | 2023 | *Outcome not investigated* | During hospital stay | *Outcome not investigated* | *Outcome not investigated* | *Outcome not investigated* |
| Conradi | 2012 | *Outcome not investigated* | 30 days | *Outcome not investigated* | *Outcome not investigated* | *Outcome not investigated* |
| Deharo | 2020 | *Outcome not investigated* | 30 days | At final follow-up (Mean: 790 ± 751 days; IQR: 79-1.310 days) | 30 days | At final follow-up (Mean: 790 ± 751 days; IQR: 79-1.310 days) |
| Demal | 2022 | Within 72 hours | 30 days | *Outcome not investigated* | *Outcome not investigated* | *Outcome not investigated* |
| Dokollari | 2021 | *Outcome not investigated* | 30 days | *Outcome not investigated* | *Outcome not investigated* | *Outcome not investigated* |
| Ejiofor | 2016 | During the operation | Procedural data used | 1 year (derived from Kaplan-Meier curve) | *Outcome not investigated* | *Outcome not investigated* |
| Erlebach | 2015 | *Outcome not investigated* | 30 days | 1 year | *Outcome not investigated* | *Outcome not investigated* |
| Fedorov | 2023 | *Outcome not investigated* | 30 days | 1 year | *Outcome not investigated* | *Outcome not investigated* |
| Gatta | 2023 | *Outcome not investigated* | During hospital stay | *Outcome not investigated* | *Outcome not investigated* | *Outcome not investigated* |
| Grubitzsch | 2017 | In 0-3 days | 30 days | 1 year | *Outcome not investigated* | 1 year |
| Hecht | 2022 | *Outcome not investigated* | 30 days | 1 year | *Outcome not investigated* | *Outcome not investigated* |
| Hernandez-Vaquero | 2019 | *Outcome not investigated* | 30 days (derived from Kaplan-Meier curve) | *Outcome not investigated* | *Outcome not investigated* | *Outcome not investigated* |
| Hirji | 2020 | *Outcome not investigated* | 30 days | *Outcome not investigated* | *Outcome not investigated* | *Outcome not investigated* |
| Majmundar | 2021 | *Outcome not investigated* | 30 days | *Outcome not investigated* | *Outcome not investigated* | *Outcome not investigated* |
| Malik | 2020 | Undefined | During hospital stay | *Outcome not investigated* | *Outcome not investigated* | *Outcome not investigated* |
| Patel | 2021 | *Outcome not investigated* | During hospital stay OR 30 days | *Outcome not investigated* | *Outcome not investigated* | *Outcome not investigated* |
| Santarpino | 2016 | Undefined | During hospital stay | *Outcome not investigated* | During hospital dtay | *Outcome not investigated* |
| Sedeek | 2019 | Undefined | Procedural data used | At final follow-up (Mean: 2.1 years; IQR: 1.2-4.2 years). [Thus excluded] | *Outcome not investigated* | *Outcome not investigated* |
| Silaschi | 2017 | Within 72 hours | 30 days | *Outcome not investigated* | 30 days | *Outcome not investigated* |
| Spaziano | 2017 | *Outcome not investigated* | 30 days | 1 year | *Outcome not investigated* | *Outcome not investigated* |
| Stankowski | 2020 | During the operation | 30 days | At final follow-up (Mean 5.6 years; Range: 12 months–16 years). | *Outcome not investigated* | *Outcome not investigated* |
| Tam | 2020 | *Outcome not investigated* | 30 days | 1 year (derived from Kaplan-Meier curve) | *Outcome not investigated* | *Outcome not investigated* |
| van Steenbergen | 2021 | From start of procedure up to first three post-operative days | 30 days | 1 year | *Outcome not investigated* | *Outcome not investigated* |
| Vukadinovikj | 2021 | Within 72 hours | 30 days | *Outcome not investigated* | *Outcome not investigated* | *Outcome not investigated* |
| Woitek | 2020 | *Outcome not investigated* | 30 days (derived from Kaplan-Meier curve) | 1 year (derived from Kaplan-Meier curve) | 30 days (derived from Kaplan-Meier curve) | 1 year (derived from Kaplan-Meier curve) |
| Yousef | 2023 | STS definition | *Outcome not investigated* | 1 year | *Outcome not investigated* | *Outcome not investigated* |

Supplementary Table 1: Time periods used by each study to define each primary outcome. “Undefined” indicates that a definition for the given outcome was not reported in the study; “Outcome not investigated” indicates that the study did not investigate the outcome in question.

**Supplementary Table 2**

| Leading author | Year | 30-day Stroke/TIA | 30-day MI | 30-day AKI | 30-day bleeding events | 30-day new pacemaker |
| --- | --- | --- | --- | --- | --- | --- |
| Choi | 2021 | VARC-2 | VARC-2 | VARC-2 | VARC-2 | Reported |
| Cizmic | 2023 | VARC-2 | *Outcome not investigated* | All stages | *Outcome not investigated* | Reported |
| Conradi | 2012 | Undefined. within 30 days | *Outcome not investigated* | *Outcome not investigated* | *Outcome not investigated* | Reported |
| Deharo | 2020 | VARC-2 | VARC-2 | *Outcome not investigated* | VARC-2  Major or life threatening | Reported |
| Demal | 2022 | VARC-2 | VARC-2 | Stage II or III. VARC-2 | Life-threatening or disabling. VARC-2 | Reported |
| Dokollari | 2021 | Undefined | *Outcome not investigated* | Undefined | *Outcome not investigated* | Reported |
| Ejiofor | 2016 | Undefined | *Outcome not investigated* | New onset renal failure | *Outcome not investigated* | Reported |
| Erlebach | 2015 | VARC-2 | VARC-2 | Post-procedural new dialysis | *Outcome not investigated* | Reported |
| Fedorov | 2023 | VARC-2 | VARC-2 | VARC-2 | VARC-2. major or disabling | Reported |
| Gatta | 2023 | Undefined | Undefined | Undefined | *Outcome not investigated* | Reported |
| Grubitzsch | 2017 | VARC-2 | VARC-2 | All stages reported. stage II and III extracted. VARC-2 | VARC-2. life threatening or major | Reported |
| Hecht | 2022 | Undefined | Undefined | Dialysis | *Outcome not investigated* | Reported |
| Hernandez-Vaquero | 2019 | *Outcome not investigated* | *Outcome not investigated* | *Outcome not investigated* | *Outcome not investigated* | *Outcome not investigated* |
| Hirji | 2020 | ICD-9-CM and ICD-10-CM | *Outcome not investigated* | ICD-9-CM and ICD-10-CM | ICD-9-CM and ICD-10-CM | Reported |
| Majmundar | 2021 | ICD-10-CM | *Outcome not investigated* | *Outcome not investigated* | Composite of GI bleeding, postoperative bleeding, GU bleeding, epistaxis, haemoptysis, intracranial haemorrhage, haemoperitoneum, other haemorrhages, blood transfusion, or vascular complications | Reported |
| Malik | 2020 | *Outcome not investigated* | ICD-9-CM and ICD-10-CM | ICD-9-CM and ICD-10-CM | ICD-9-CM and ICD-10-CM | Reported |
| Patel | 2021 | Undefined | Undefined | Dialysis | Undefined | Reported |
| Santarpino | 2016 | Undefined | Undefined | Dialysis | Undefined | Reported |
| Sedeek | 2019 | STS or VARC-2. reporting unclear | *Outcome not investigated* | RIFLE. I-III | Life-threatening or disabling; VARC-2 | Reported |
| Silaschi | 2017 | VARC-2 | VARC-2 | Stage II or III. VARC-2 | VARC-2 | Reported |
| Spaziano | 2017 | VARC-2 | VARC-2 | Renal failure recquiring dialysis | *Outcome not investigated* | Reported |
| Stankowski | 2020 | VARC-2 | VARC-2 | New dialysis | *Outcome not investigated* | Reported |
| Tam | 2020 | *Outcome not investigated* | *Outcome not investigated* | *Outcome not investigated* | *Outcome not investigated* | Reported |
| van Steenbergen | 2021 | Undefined | *Outcome not investigated* | *Outcome not investigated* | *Outcome not investigated* | Reported |
| Vukadinovikj | 2021 | VARC-2 | VARC-2 | Stage I-III. VARC-2 | Life-threatening or major, VARC-2 | Reported |
| Woitek | 2020 | VARC-2 | VARC-2 | Stage II or III VARC-2 | Life-threatening, VARC-2 | Reported |
| Yousef | 2023 | Undefined | Reintervention for myocardial infarction | New onset renal failure requiring dialysis | Reoperation for bleeding | Reported |

Supplementary Table 2: Definitions used by each study for secondary outcomes. “Undefined” indicates that a definition for the given outcome was not reported in the study; “Outcome not investigated” indicates that the study did not investigate the outcome in question. Some definitions utilize classifications and systems such as the International Classification of Diseases (ICD); Risk, Injury, Failure, Loss, and End-stage renal disease classification (RIFLE); and classifications proposed by the Valve Academic Research Consortium (VARC-2 or VARC-3) or the Society of Thoracic Surgeons (STS).

**Supplementary Table 3**

|  |  | Procedural mortality | | | Short-term all-cause mortality | | | Long-term all-cause mortality | | | Short-term cardiovascular mortality | | | Long-term cardiovascular mortality | | |
| --- | --- | --- | --- | --- | --- | --- | --- | --- | --- | --- | --- | --- | --- | --- | --- | --- |
| Leading author | **Year** | **ViV-TAVI** | **re-do sAVR** | **P-value** | **ViV-TAVI** | **re-do sAVR** | **P-value** | **ViV-TAVI** | **re-do sAVR** | **P-value** | **ViV-TAVI** | **re-do sAVR** | **P-value** | **ViV-TAVI** | **re-do sAVR** | **P-value** |
| Choi | 2021 | — | — | — | 3(4.11) | 7(7) | 0.523 | 1(1.37) | 5(5) | 0.407 | 1(1.37) | 1(1) | 1 | 0(0) | 0(0) |  |
| Cizmic | 2023 | — | — | — | 0(0) | 3(17.65) | <0.001 | — | — | — | — | — | — | — | — | — |
| Conradi | 2012 | — | — | — | 6(7.32) | 7(8.54) | 1.00 | — | — | — | — | — | — | — | — | — |
| Deharo | 2020 | 2(0.28) | 0(0) | — | 26(3.63) | 52(7.25) | 0.003 | 170(23.71) | 147(20.5) | 0.23 | 21(2.93) | 47(6.56) | 0.002 | 21(2.93) | 47(6.56) | 0.002 |
| Demal | 2022 | 3(1.44) | 0(0) | 0.331 | 8(3.83) | 2(3.57) | 0.778 | — | — | — | — | — | — | — | — | — |
| Dokollari | 2021 | — | — | — | 0(0) | 4(7.02) | 0.08 | — | — | — | — | — | — | — | — | — |
| Ejiofor | 2016 | 0(0) | 1(4.55) | 1.000 | 0(0) | 1(4.55) | 1.000 | 5(22.73) | 6(27.27) | — | — | — | — | — | — | — |
| Erlebach | 2015 | — | — | — | 2(4) | 0(0) | 0.238 | 8(16) | 2(3.85) | <0.001 | — | — | — | — | — | — |
| Fedorov | 2023 | — | — | — | 0(0) | 0(0) | — | 1(2.7) | 0(0) | — | — | — | — | — | — | — |
| Gatta | 2023 | — | — | — | 0(0) | 9(7.2) | 0.002 | — | — | — | — | — | — | — | — | — |
| Grubitzsch | 2017 | 2(7.41) | 2(8) | — | 3(11.11) | 2(8) | 1.0 | 5(18.52) | 4(16) | — |  |  | — | 4(14.81) | 3(12) | — |
| Hecht | 2022 | — | — | — | 2(2.5) | 9(8.65) | 0.10 | 6(7.5) | 13(12.5) | — | — | — | — | — | — | — |
| Hernandez-Vaquero | 2019 | — | — | — | 4(7.02) | 2(3.51) | — | — | — | — | — | — | — | — | — | — |
| Hirji | 2020 | — | — | — | 61(2.8) | 109(5) | 0.018 | — | — | — | — | — | — | — | — | — |
| Majmundar | 2021 | — | — | — | 35(0.95) | 8(0.27) | 0.471 | — | — | — | — | — | — | — | — | — |
| Malik | 2020 | 6(0.85) | 35(4.93) | 0.07 | 7(0.99) | 35(4.93) | 0.06 | — | — | — | — | — | — | — | — | — |
| Patel | 2021 | — | — | — | 3(1.6) | 1(1.16) | 0.92 | — | — | — | — | — | — | — | — | — |
| Santarpino | 2016 | 0(0) | 0(0) | — | 0(0) | 0(0) | — | — | — | — | 0(0) | 0(0) | — |  |  | — |
| Sedeek | 2019 | 2(2.22) | 7(2.69) | 1.000 | 2(2.22) | 7(2.69) | 1.000 | 19(21.11) | 49(18.85) | — | — | — | — | — | — | — |
| Silaschi | 2017 | 1(1.41) | 1(1.69) | 1.00 | 3(4.23) | 3(5.08) | 1.00 | — | — | — | 2(2.82) | 3(5.08) | 0.66 |  |  |  |
| Spaziano | 2017 | — | — | — | 3(3.85) | 5(6.41) | 0.49 | 9(11.54) | 10(12.82) | 0.80 | — | — | — | — | — | — |
| Stankowski | 2020 | 1(1.47) | 0(0) | — | 1(3.34) | 3(10.00) | — | 21(30.88) | 14(35) | — | — | — | — | — | — | — |
| Tam | 2020 | — | — | — | — | — | — | 8(6.11) | 19(14.50) | — | — | — | — | — | — | — |
| van Steenbergen | 2021 | 2(1.21) | 3(1.82) | 0.42 | 5(3.03) | 10(6.06) | 0.40 | 17(10.3) | 17(10.30) | 0.48 | — | — | — | — | — | — |
| Vukadinovikj | 2021 | — | — | — | 3(12) | 0(0) | 0.542 | — | — | — | — | — | — | — | — | — |
| Woitek | 2020 | — | — | — | 7(4.76) | 5(4.50) | — | 13(8.84) | 11(9.91) | — | 6(4,08) | 4(3,60) | — | 12(8.16) | 9(8.11) | — |
| Yousef | 2023 | 4(2.02) | 3(2.04) | 0.99 | — | — | — | 56(28.28) | 69(46.94) | — | — | — | — | — | — | — |

Supplementary Table 3: Extracted mortality data including procedural mortality; short-term all-cause mortality; long-term all-cause mortality; short-term mortality resulting from cardiovascular (CV) causes; and long-term mortality resulting from cardiovascular (CV) causes. All data are reported as n(%).

**Supplementary Table 4**

|  |  | Population Age | | | Female sex | | | Diabetic | | | Hypertension | | | Smoking | | | Dyslipidaemia | | |
| --- | --- | --- | --- | --- | --- | --- | --- | --- | --- | --- | --- | --- | --- | --- | --- | --- | --- | --- | --- |
| Leading Author | **Year** | **ViV-TAVI** | **re-do SAVR** | **P-value** | **ViV-TAVI** | **re-do SAVR** | **P-value** | **ViV-TAVI** | **re-do SAVR** | **P-value** | **ViV-TAVI** | **re-do SAVR** | **P-value** | **ViV-TAVI** | **re-do SAVR** | **P-value** | **ViV-TAVI** | **re-do SAVR** | **P-value** |
| Choi | 2021 | 66.57 ± 13.44 | 58.03 ± 13.86 | <0.0001 | 18(24.66) | 28(28) | 0.623 | 19(26.03) | 27(27) | 0.886 | 50(68.49) | 68(68) | 0.9452 | 16(21.92) | 47(47) | 0.0007 | 34(46.58) | 39(39) | 0.3191 |
| Cizmic | 2023 | 78 ± 7.4 | 62.1 ± 16.2 | 0.012 | 41(56.2) | 6(35.3) | 0.121 | 31(42.5) | 2(11.8) | 0.018 | 70(95.9) | 9(52.7) | <0.001 | 7(9.6) | 4(23.5) | 0.114 | 48(65.8) | 5(29.4) | 0.006 |
| Conradi | 2012 | 81.9 ± 5.2 | 82.5 ± 4.1 | 0.39 | 52(63.4) | 48(58.5) | 0.52 | 28(34.2) | 25(30.5) | 0.74 | 68(82.9) | 73(89) | 0.27 | **—** | **—** | **—** | **—** | **—** | **—** |
| Deharo | 2020 | 74.9 ± 9.7 | 74.5 ± 8.2 | 0.33 | 315(43.9) | 303(42.3) | 0.52 | 227(31.7) | 217(30.3) | 0.57 | 569(79.4) | 558(77.8) | 0.48 | 99(13.8) | 109(15.2) | 0.45 | 388(54.1) | 379(52.9) | 0.63 |
| Demal | 2022 | 77.9 ± 7 | 71 ± 6.6 | <0.001 | **—** | **—** | **—** | 37(19.1) | 10(15.4) | 0.504 | **—** | **—** | **—** | **—** | **—** | **—** | **—** | **—** | **—** |
| Dokollari | 2021 | 79.06 ± 7.4 | 67.19 ± 14.12 | <0.01 | 14(45.2) | 28(49.1) | 0.83 | 7(22.6) | 16(28.1) | 0.79 | 28(90.3) | 47(82.5) | 0.49 | 7(22.6) | 23(40.4) | 0.14 | 27(87.1) | 42(73.7) | 0.23 |
| Ejiofor | 2016 | 75 ± 9.6 | 74.5 ± 10.4 | 0.749 | 8(36.4) | 9(40.9) | 1.000 | 10(45.45) | 5(22.73) | 0.203 | 21(95.5) | 20(95.5) | 1.000 | **—** | **—** | **—** | **—** | **—** | **—** |
| Erlebach | 2015 | 78.1 ± 6.7 | 66.2 ± 13.1 | <0.001 | 23(46) | 14(27) | 0.064 | 10(20) | 5(10) | 0.169 | 41(82) | 38(73) | 0.346 | **—** | **—** | **—** | **—** | **—** | **—** |
| Fedorov | 2023 | 75 ± 8 | 62 ± 11.4 | 0.0001 | 12(32.4) | 1(5.3) | 0.04 | 6(16.2) | 2(10.5) | 0.7 | 35(94.6) | 15(78.9) | 0.17 | **—** | **—** | **—** | **—** | **—** | **—** |
| Gatta | 2023 | 75.2 ± 8.2 | 74.8 ± 8.8 | 0.7 | 52(41.6) | 46(36.8) | 0.4 | 30(24) | 26(20.8) | 0.5 | 94(75.2) | 98(78.4) | 0.5 | **—** | **—** | **—** | **—** | **—** | **—** |
| Grubitzsch | 2017 | 75.3 ± 9.9 | 69.0 ± 8.6 | 0.060 | **—** | **—** | **—** | **—** | **—** | **—** | **—** | **—** | **—** | **—** | **—** | **—** | **—** | **—** | **—** |
| Hecht | 2022 | 74.4 ± 10.9 | 70.3 ± 7.3 | 0.005 | 27(33.8) | 38(36.5) | 0.695 | 28(35) | 35(33.7) | 0.849 | 67(83.8) | 85(81.7) | 0.720 | **—** | **—** | **—** | **—** | **—** | **—** |
| Hernandez-Vaquero | 2019 | 79.82 | 78.81 | **—** | 29(50.9) | 35(61.4) | **—** | **—** | **—** | **—** | **—** | **—** | **—** | **—** | **—** | **—** | **—** | **—** | **—** |
| Hirji | 2020 | 72.5 ± 12 | 72.9 ± 12.2 | 0.316 | 848(38.9) | 833(38.2) | 0.709 | 604(27.7) | 548(25.1) | 0.161 | 1228(56.3) | 1214(55.7) | 0.761 | — | — | **—** | 1265(58) | 1244(57) | 0.641 |
| Majmundar | 2021 | 79 ± 7.8 | 65 ± 11.2 | <0.001 | 1820(48.9) | 1185(38.9) | <0.001 | 1350(36.3) | 905(29.7) | <0.001 | 3289(88.3) | 2527(83) | <0.001 | 1439(38.6) | 1113(36.5) | 0.229 | 2641(70.9) | 1930(63.4) | <0.001 |
| Malik | 2020 | 73.7 ± 10.4 | 73.3 ± 8.6 | 0.7279 | 335.12(47.2) | 320.21(45.1) | 0.7129 | 235.01(33.1) | 230.04(32.4) | 0.8963 | 590.01(83.1) | 555.22(78.2) | 0.8963 | **—** | **—** | **—** | **—** | **—** | **—** |
| Patel | 2021 | 73 ± 13.1 | 61.3 ± 14.8 | <0.001 | 60(32.1) | 29(33.7) | 0.79 | 73(39) | 30(34.9) | 0.51 | 175(93.6) | 72(83.7) | 0.01 | **—** | **—** | **—** | **—** | **—** | **—** |
| Santarpino | 2016 | **—** | **—** | **—** | **—** | **—** | **—** | **—** | **—** | **—** | **—** | **—** | **—** | **—** | **—** | **—** | **—** | **—** | **—** |
| Sedeek | 2019 | 79 | 72 | <.001 | 17(19) | 83(32) | 0.018 | 25(28) | 57(22) | 0.258 | 79(88) | 191(73) | 0.005 | **—** | **—** | **—** | **—** | **—** | **—** |
| Silaschi | 2017 | 78.6 ± 7.5 | 72.9 ± 6.6 | <0.01 | 30(42.3) | 23(39) | 0.72 | 8(11.3) | 6(10.2) | 1.00 | **—** | **—** | **—** | **—** | **—** | **—** | **—** | **—** | **—** |
| Spaziano | 2017 | 78.0±8.0 | 77.4±5.0 | 0.58 | 39 (50) | 34 (44) | 0.52 | 15 (19) | 12 (15) | 0.67 | 56(72) | 57(73) | 1 | **—** | **—** | **—** | **—** | **—** | **—** |
| Stankowski | 2020 | 79.2 ± 68 | 72.9 ± 7.2 | **—** | 40(58.8) | 15(37.5) | **—** | 23(33.8) | 18(45) | **—** | 63(92.6) | 36(90) | **—** | 6(8.8) | 2(5) | **—** | **—** | **—** | **—** |
| Tam | 2020 | 75.9 ± 8.8 | 76.11 ± 6.32 | 0.82 | 50(38.2) | 50(38.2) | 1.00 | 65(49.6) | 62(47.3) | 0.81 | 120(91.6) | 116(88.5) | 0.54 | 60(45.802) | 61(46.565) | **—** | 96(73.3) | 91(69.5) | 0.59 |
| van Steenbergen | 2021 | 74 | 73 | 0.11 | 69(41.8) | 65(39.4) | 0.74 | 34(20.6) | 29(17.6) | 0.58 | **—** | **—** | **—** | **—** | **—** | **—** | **—** | **—** | **—** |
| Vukadinovikj | 2021 | 75.4 ± 1.7 | 62.9 ± 5.1 | 0.019 | 5(20) | 2(20) | >0.999 | **—** | **—** | **—** | **—** | **—** | **—** | **—** | **—** | **—** | **—** | **—** | **—** |
| Woitek | 2020 | 76.2 ± 8 | 58.5 ± 14.4 | **—** | 55(40.1) | 45(37.4) | **—** | 53(36.1) | 18(16.2) | **—** | 144(98) | 96(86.5) | **—** | **—** | **—** | **—** | **—** | **—** | **—** |
| Yousef | 2023 | 79.5 | 65 | **<.001** | 84(42.4) | 50(34) | 0.11 | 83(41.9) | 48(32.6) | 0.08 | 178(89.9) | 125(85) | 0.17 | **—** | **—** | **—** | **—** | **—** | **—** |

Supplementary Table 4: Extracted baseline characteristics of the study populations including population age, female sex, diabetes, hypertension, smoking, and dyslipidaemia. Age data presented as mean ± standard deviation (or as mean only, when standard deviation was not provided), all other data presented as n(%).

**Supplementary Table 5**

|  |  | Pre-op EF | | | Post-op EF | | | Pre-op AVG | | | Post-op AVG | | | Post-op AVG >20 mmHg | | |
| --- | --- | --- | --- | --- | --- | --- | --- | --- | --- | --- | --- | --- | --- | --- | --- | --- |
| Leading author | **Year** | **ViV-TAVI** | **re-do sAVR** | **P-value** | **ViV-TAVI** | **re-do sAVR** | **P-value** | **ViV-TAVI** | **re-do sAVR** | **P-value** | **ViV-TAVI** | **re-do sAVR** | **P-value** | **ViV-TAVI** | **re-do sAVR** | **P-value** |
| Choi | 2021 | — | — | — | — | — | — | — | — | — | — | — | — | — | — | — |
| Cizmic | 2023 | 51.4 ± 12 | 51.1 ± 12 | 0.224 | 51.9 ± 11.9 | 51.6 ± 13 | 0.870 | — | — | — | 17.2 ± 10.1 | 11 ± 6.6 | 0.503 | — | — | — |
| Conradi | 2012 | 52.5 ± 8.4 | 50.6 ± 10.7 | 0.23 | — | — | — | 65 ± 24.9 | 75.3 ± 25.5 | 0.01 | 11.3 ± 5.6 | 11.8 ± 5.3 | 0.62 | — | — | — |
| Deharo | 2020 | — | — | — | — | — | — | — | — | — | — | — | — | — | — | — |
| Demal | 2022 | — | — | — | — | — | — | 30.8 ± 16.2 | 33 ± 13.3 | 0.389 | 15.8 ± 7.5 | 13.1 ± 8.2 | 0.028 | 50(23.92) | 4(6.15) | <0.001 |
| Dokollari | 2021 | 49 ± 14.01 | 50.46 ± 12.84 | 0.62 | 45 ± 14.4 | 47.1 ± 15 | 0.42 | 29.6 ± 18.4 | 39.5 ± 20.3 | 0.02 | 16.8 ± 1 | 16.2 ± 7.2 | 0.72 | — | — | — |
| Ejiofor | 2016 | 55 | 55 | 0.221 | — | — | — | 39.8 ± 13.6 | 46.6 ± 26.5 | 0.001 | 12.4 ± 6.2 | 13.5 ± 13.2 | 0.584 | 2(10) | 0(0) | — |
| Erlebach | 2015 | 49.8 ± 13.1 | 56.7 ± 15.8 | 0.019 | — | — | — | — | — | — | 18.8 ± 8.7 | 13.8 ± 5.4 | 0.008 | — | — | — |
| Fedorov | 2023 | 55.7 ± 10.9 | 53.5 ± 10.1 | 0.47 | 54.9 ± 9.9 | 57.5 ± 12.4 | 0.39 | 15.5 ± 10.1 | 15.1 ± 15.1 | 0.9 | 13.2 ± 4.3 | 11.5 ± 3.5 | 0.20 | — | — | — |
| Gatta | 2023 | — | — | — | — | — | — | 39.5 ± 22 | 34.7 ± 20.4 | 0.1 | 17.6 ± 9.7 | 11.9 ± 4.6 | <0.001 | — | — | — |
| Grubitzsch | 2017 | — | — | — | — | — | — | — | — | — | 11.8 ± 3.2 | 15.9 ± 3.5 | — | 1(3.70) | 5(20) | 0.2 |
| Hecht | 2022 | — | — | — | — | — | — | — | — | — | — | — | — | — | — | — |
| Hernandez-Vaquero | 2019 | 60 | 60 | — | — | — | — | — | — | — | — | — | — | — | — | — |
| Hirji | 2020 | — | — | — | — | — | — | — | — | — | — | — | — | — | — | — |
| Majmundar | 2021 | — | — | — | — | — | — | — | — | — | — | — | — | — | — | — |
| Malik | 2020 | — | — | — | — | — | — | — | — | — | — | — | — | — | — | — |
| Patel | 2021 | 48.7 ± 13.4 | 53.4 ± 10.7 | 0.005 | 47.8 ± 14.3 | 53.8 ± 10.5 | 0.02 | 39 ± 17.9 | 43 ± 18.1 | 0.11 | 16.6 ± 9 | 14.3 ± 7.9 | 0.08 | — | — | — |
| Santarpino | 2016 | — | — | — | — | — | — | — | — | — | — | — | — | — | — | — |
| Sedeek | 2019 | — | — | — | — | — | — | — | — | — | 19 | 15 | <0.001 | — | — | — |
| Silaschi | 2017 | — | — | — | — | — | — | 33 ± 17.8 | 37.3 ± 13.7 | 0.23 | 19.7 ± 7.7 | 12.2 ± 5.7 | <0.01 | 33(46.48) | 3(5.08) | <0.01 |
| Spaziano | 2017 | 50.7 ± 13.5 | 49.5 ± 13.4 | 0.58 | — | — | — | — | — | — | 18.1 ± 7.4 | 14.3 ± 6.2 | 0.01 | 21(26.92) | 7(8.97) | 0.04 |
| Stankowski | 2020 | 52.1 ± 10.7 | 56.3 ± 8.7 | 0.035 | 52.4 ± 9.4 | 53.1 ± 6 | 0.675 | 41.9 ± 19.3 | 40.1 ± 20.9 | 0.646 | 16.8 ± 8.6 | 19 ± 11.3 | 0.278 | — | — | — |
| Tam | 2020 | — | — | — | — | — | — | — | — | — | — | — | — | — | — | — |
| van Steenbergen | 2021 | — | — | — | — | — | — | — | — | — | — | — | — | — | — | — |
| Vukadinovikj | 2021 | 51.7 ± 2.2 | 51.9 ± 4.9 | 0.797 | — | — | — | — | — | — | — | — | — | — | — | — |
| Woitek | 2020 | 54.5 ± 13.9 | 57.4 ± 10.2 | — | — | — | — | — | — | — | 17.4 ± 8.5 | 11.9 ± 5 | — | — | — | — |
| Yousef | 2023 | 55 | 58 | 0.02 | 56.5 | 57.5 | 0.39 | 41 | 45.5 | 0.03 | 13 | 8.8 | <0.001 | — | — | — |

Supplementary Table 5: Extracted echocardiographic data including mean pre-operative (i.e. baseline) ejection fraction (Pre-op EF), mean post-operative ejection fraction (Post-op EF), mean pre-operative aortic valve gradient (Pre-op AVG), mean post-operative aortic valve gradient (Post-op AVG) and number of patients with a mean post-operative aortic valve gradient >20mmHg (Post-Op AVG >20mmHg). EF and AVG are presented as mean ± standard deviation (or as mean only, when standard deviation was not provided). Post-op AVG > 20mmHg presented as n(%).

**Supplementary Table 6**

|  |  | Cross clamp time (mins) | CBP time (mins) | Duration (mins) |  |
| --- | --- | --- | --- | --- | --- |
| Leading author | **Year** | **re-do sAVR only** | **re-do sAVR only** | **ViV-TAVI** | **re-do sAVR** |
| Choi | 2021 | — | — | 212.97 ± 85.77 | 848.604 ± 342.49 |
| Cizmic | 2023 | 71.8 ± 18.1 | 118.2 ± 38 | 90.8 ± 35 | 220.7 ± 47.2 |
| Conradi | 2012 | 70.8 ± 23.2 | 111 ± 36.2 | 128.9 ± 9 | 200.9 ± 36.2 |
| Deharo | 2020 | — | — | 111.8 ± 56.5 | 262.9 ± 86 |
| Demal | 2022 | 85.9 ± 27.9 | 143.8 ± 70.8 | — | — |
| Dokollari | 2021 | 88.3 ± 34.4 | 109.9 ± 40.9 | 85 ± 24.8 | 251.2 ± 80.7 |
| Ejiofor | 2016 | 90 | 138 | — | — |
| Erlebach | 2015 | 78.7 ± 19.4 | 110.3 ± 28.8 | — | — |
| Fedorov | 2023 | 76.6 ± 30.2 | 110.7 ± 38.3 | 61.2 ± 18.2 | 228.6 ± 50.9 |
| Gatta | 2023 | 91.3 ± 36.3 | 142.2 ± 62.4 | — | — |
| Grubitzsch | 2017 | 101 ± 25.3 | 125 ± 36.3 | — | — |
| Hecht | 2022 | — | — | — | — |
| Hernandez-Vaquero | 2019 | — | — | — | — |
| Hirji | 2020 | — | — | — | — |
| Majmundar | 2021 | — | — | — | — |
| Malik | 2020 | — | — | — | — |
| Patel | 2021 | — | — | — | — |
| Santarpino | 2016 | — | — | — | — |
| Sedeek | 2019 | — | — | — | — |
| Silaschi | 2017 | 79 ± 24.5 | 126.1 ± 56.6 | 100 ± 47.6 | 269.6 ± 77.4 |
| Spaziano | 2017 | — | — | — | — |
| Stankowski | 2020 | 79.8 ± 30.8 | 90 ± 37.6 | — | — |
| Tam | 2020 | — | — | — | — |
| van Steenbergen | 2021 | — | — | — | — |
| Vukadinovikj | 2021 | 136 ± 12 | 194 ± 19 | — | — |
| Woitek | 2020 | — | — | — | — |
| Yousef | 2023 | — | — | — | — |

Supplementary Table 6: Extracted perioperative data including aortic cross clamp time, time spent on cardiopulmonary bypass (CBP time), and procedure duration. All data presented as mean ± standard deviation (or as mean only, where standard deviation was not provided).

**Supplementary Table 7**

|  |  | EUROSCORE | | | | STS | | | NYHA ≥III | | |
| --- | --- | --- | --- | --- | --- | --- | --- | --- | --- | --- | --- |
| Authors | **Year** | **Type** | **ViV-TAVI** | **re-do sAVR** | **P-value** | **ViV-TAVI** | **re-do sAVR** | **P-value** | **ViV-TAVI** | **re-do sAVR** | **P-value** |
| Choi | 2021 | not specified | 7.51 ± 8.24 | 4.32 ± 2.98 | p < 0.05 | 4.68 ± 5.51 | 2.78 ± 2.09 | p < 0.01 | 57(85.08) | 6(6) | P <0.0001 |
| Cizmic | 2023 | N/A | — | — | — | 6.4 ± 3.1 | 6.4 ± 3.2 | 0.392 | — | — | — |
| Conradi | 2012 | not specified | 23.9 ± 11.5 | 23.6 ± 10.4 | 0.85 | 8.5 ± 1.3 | 9 ± 4.9 | 0.74 | 70(85.37) | 65(79.27) | — |
| Deharo | 2020 | EUROSCORE II | 4.7 ± 1 | 4.7 ± 1 | 0.46 | — | — | — | — | — | — |
| Demal | 2022 | EUROSCORE II | 10 ± 6.4 | 8 ± 4.8 | 0.021 | — | — | — | — | — | — |
| Dokollari | 2021 | EUROSCORE II | 9.46 ± 7.3 | 11.02 ± 9.33 | 0.42 | — | — | — | 31(100) | 52(91.23) | — |
| Ejiofor | 2016 | N/A | — | — | — | 7.54 ± 3 | 7.7 ± 3.4 | 0.360 | 21(95.5) | 16(72.7) | 0.095 |
| Erlebach | 2015 | not specified | 27.4 ± 18.7 | 14.4 ± 10 | <0.001 | — | — | — | 46(92) | 20(38.46) | — |
| Fedorov | 2023 | EUROSCORE II | 9.7 ± 6.3 | 4.3 ± 2.2 | < 0.0001 | — | — | — | 28(75.68) | 14(73.68) | — |
| Gatta | 2023 | not specified | 17.9 ± 11.3 | 17.8 ± 9.1 | 0.9 | — | — | — | 91(72.8) | 92(73.6) | — |
| Grubitzsch | 2017 | EUROSCORE II | 13 ± 10.4 | 8.9 ± 6.5 | 0.054 | — | — | — | — | — | — |
| Hecht | 2022 | EUROSCORE II | 7.5 | 9.3 | 0.471 | — | — | — | 54(67.5) | 68(65.4) | — |
| Hernandez-Vaquero | 2019 | EUROSCORE II | 8.34 | 11.14 | — | — | — | — | — | — | — |
| Hirji | 2020 | N/A | — | — | — | — | — | — | — | — | — |
| Majmundar | 2021 | N/A | — | — | — | — | — | — | — | — | — |
| Malik | 2020 | N/A | — | — | — | — | — | — | — | — | — |
| Patel | 2021 | N/A | — | — | — | 8.4 ± 7.6 | 5.5 ± 4.6 | 0.005 | 180(96.8) | 47(66.2) | <.001 |
| Santarpino | 2016 | N/A | — | — | — | — | — | — | — | — | — |
| Sedeek | 2019 | N/A | — | — | — | 7.5 | 3 | <.001 | 75(83) | 162(62) | <.001 |
| Silaschi | 2017 | EUROSCORE I | 25.1 ± 18.9 | 16.8 ± 9.3 | < 0.01 | — | — | — | — | — | — |
| Spaziano | 2017 | not specified | 22.1 ± 16 | 22.1 ± 18.3 | 0.99 | 7.2 ± 4.9 | 5.8 ± 4.6 | 0.09 | 63(80.77) | 65(83.33) | — |
| Stankowski | 2020 | EUROSCORE II | 10.9 ± 6.2 | 7.8 ± 4.3 | — | — | — | — | 63(92.6) | 34(85) | — |
| Tam | 2020 | N/A | — | — | — | — | — | — | — | — | — |
| van Steenbergen | 2021 | EUROSCORE I | — | — | — | — | — | — | 90(54.5) | 95(57.6) | 0.66 |
| Vukadinovikj | 2021 | EUROSCORE I | 26.8 ± 3.1 | 17.7 ± 4.6 | 0.090 | 7.4 ± 1.3 | 3.2 ± 1.2 | 0.007 | 18(72) | 4(40) | 0.123 |
| Vukadinovikj | 2021 | EUROSCORE II | 11.5 ± 1.6 | 5.6 ± 1.5 | 0.007 | 7.4 ± 1.3 | 3.2 ± 1.2 | 0.007 | 18(72) | 4(40) | 0.123 |
| Woitek | 2020 | N/A | — | — | — | 8.27 ± 6.12 | 2.76 ± 2.09 | — | 107(72.8) | 61(55.5) | — |
| Yousef | 2023 | N/A | — | — | — | 6.28 | 1.7 | <0.001 | 144(72.73) | 46(31.29) | <0.001 |

Supplementary Table 7: Extracted data pertaining to the European System for Cardiac Operative Risk Evaluation (EUROSCORE) and Society of Thoracic Surgeons score (STS) for risk of mortality and number of patients in classes III or IV of the New York Heart Association functional classification (NYHA), data presented as mean ± standard deviation (or as mean only, when standard deviation was not provided) or as n(%).
