# Supplementary figures and images for: Transcatheter aortic valve implantation vs. surgery for failed bioprosthesis: a meta-analysis of over 20 000 patients
Source: J Cardiovasc Med (Hagerstown). 2025 Jan 20;26(3):153–66. doi: 10.2459/JCM.0000000000001702 (PMC11841718; doi:10.2459/JCM.0000000000001702)

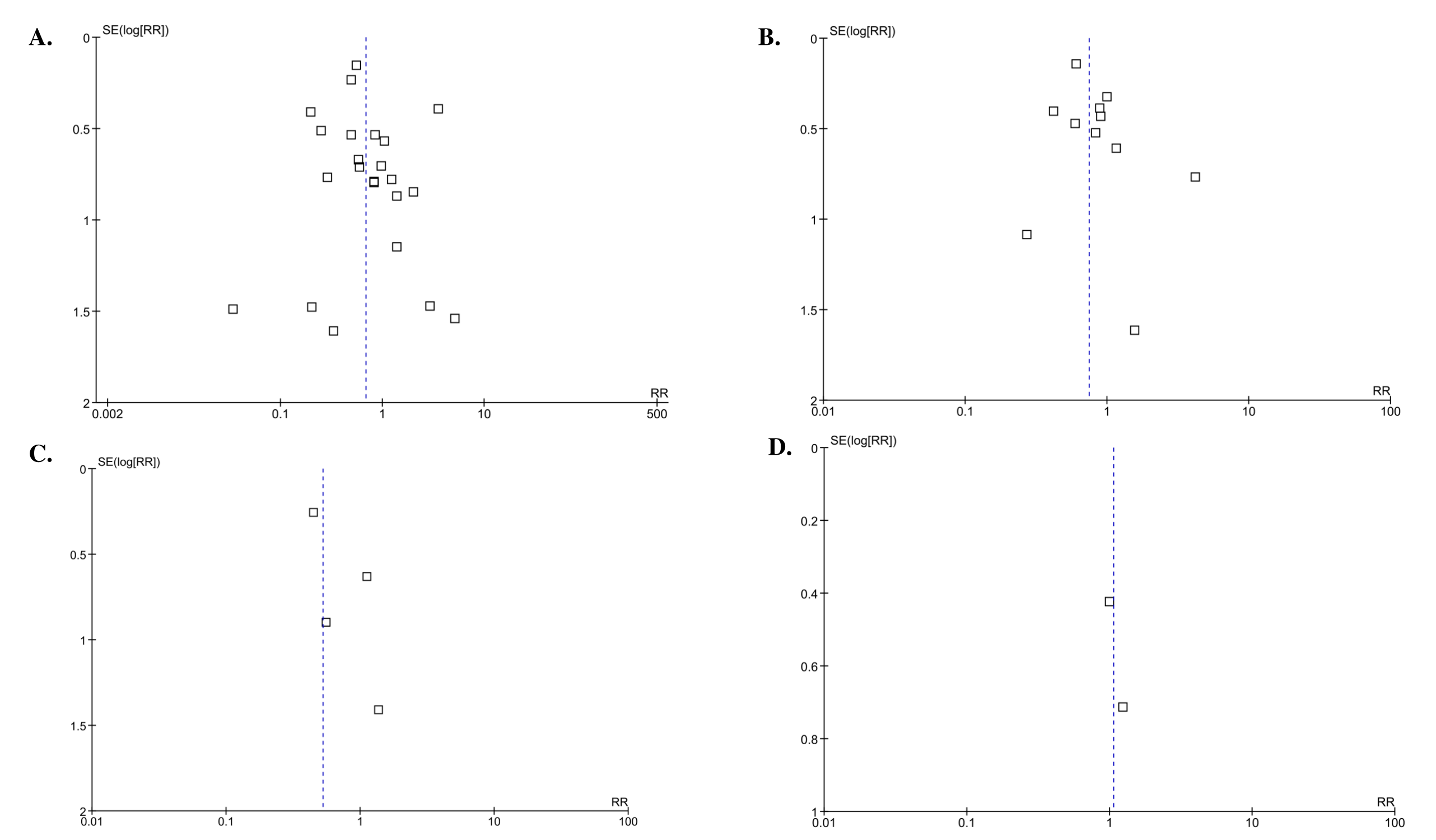

Supplement: Supplemental Digital Content [file jcarm-26-153-s003.tiff]

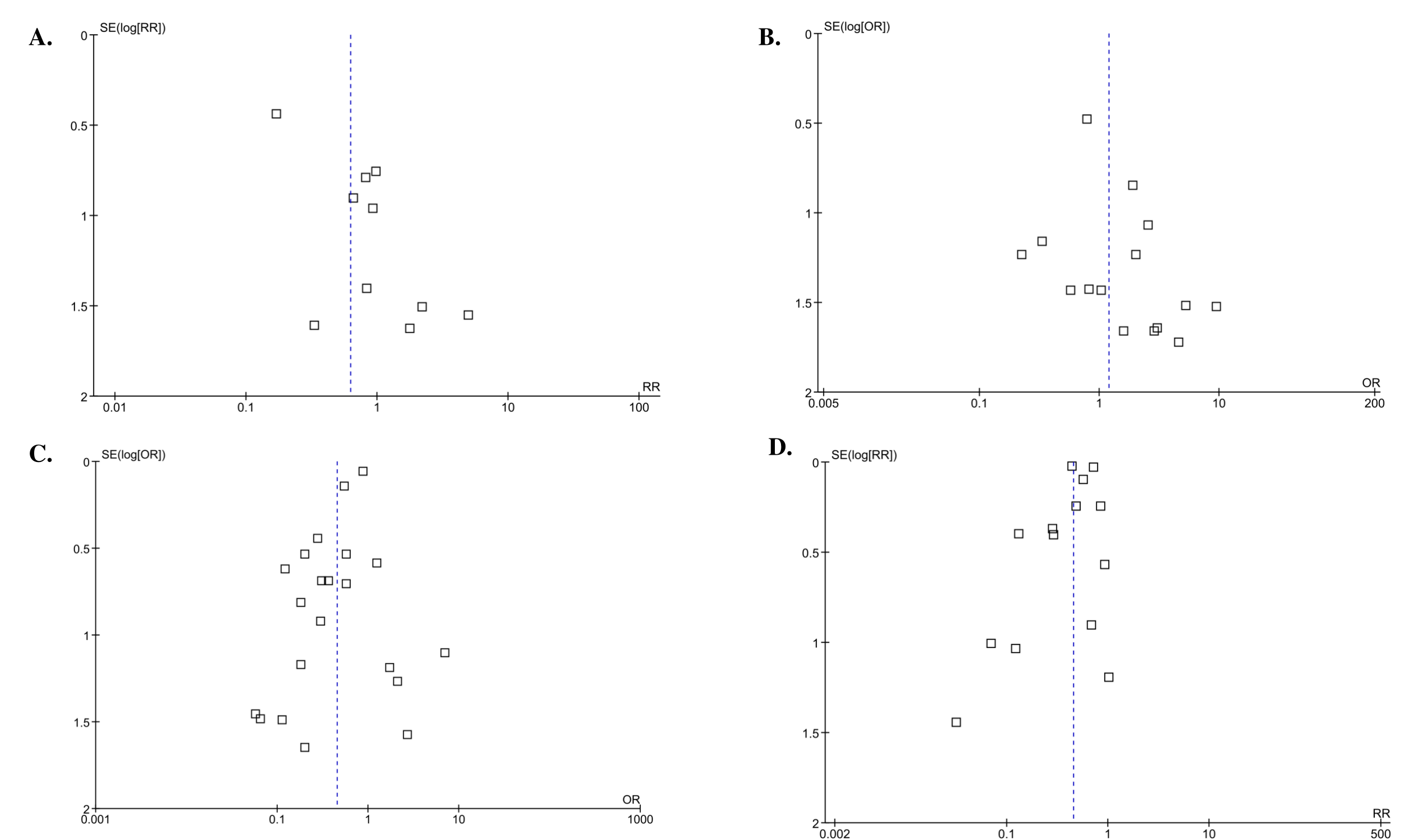

Supplement: Supplemental Digital Content [file jcarm-26-153-s004.tiff]

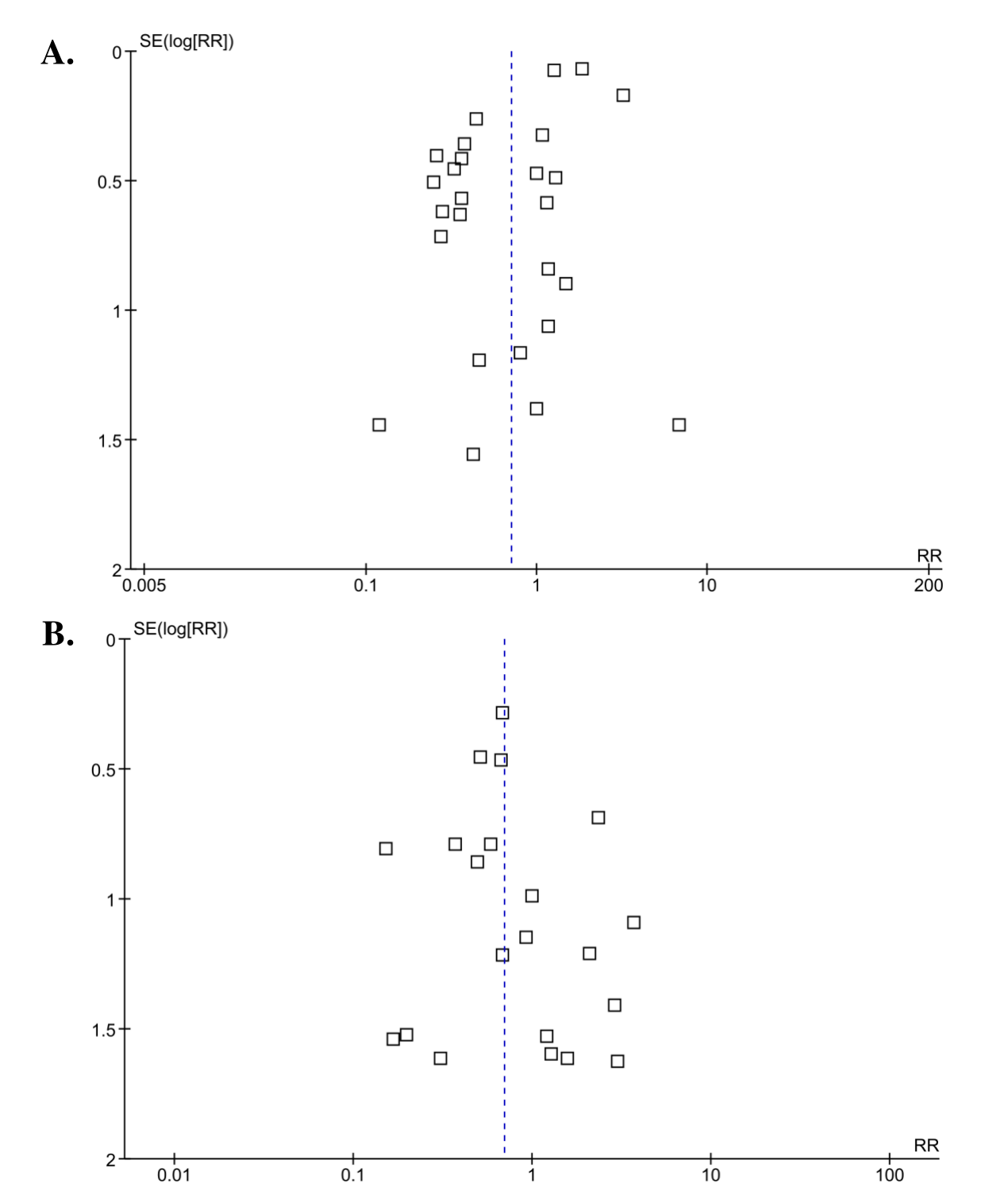

Supplement: Supplemental Digital Content [file jcarm-26-153-s005.tiff]

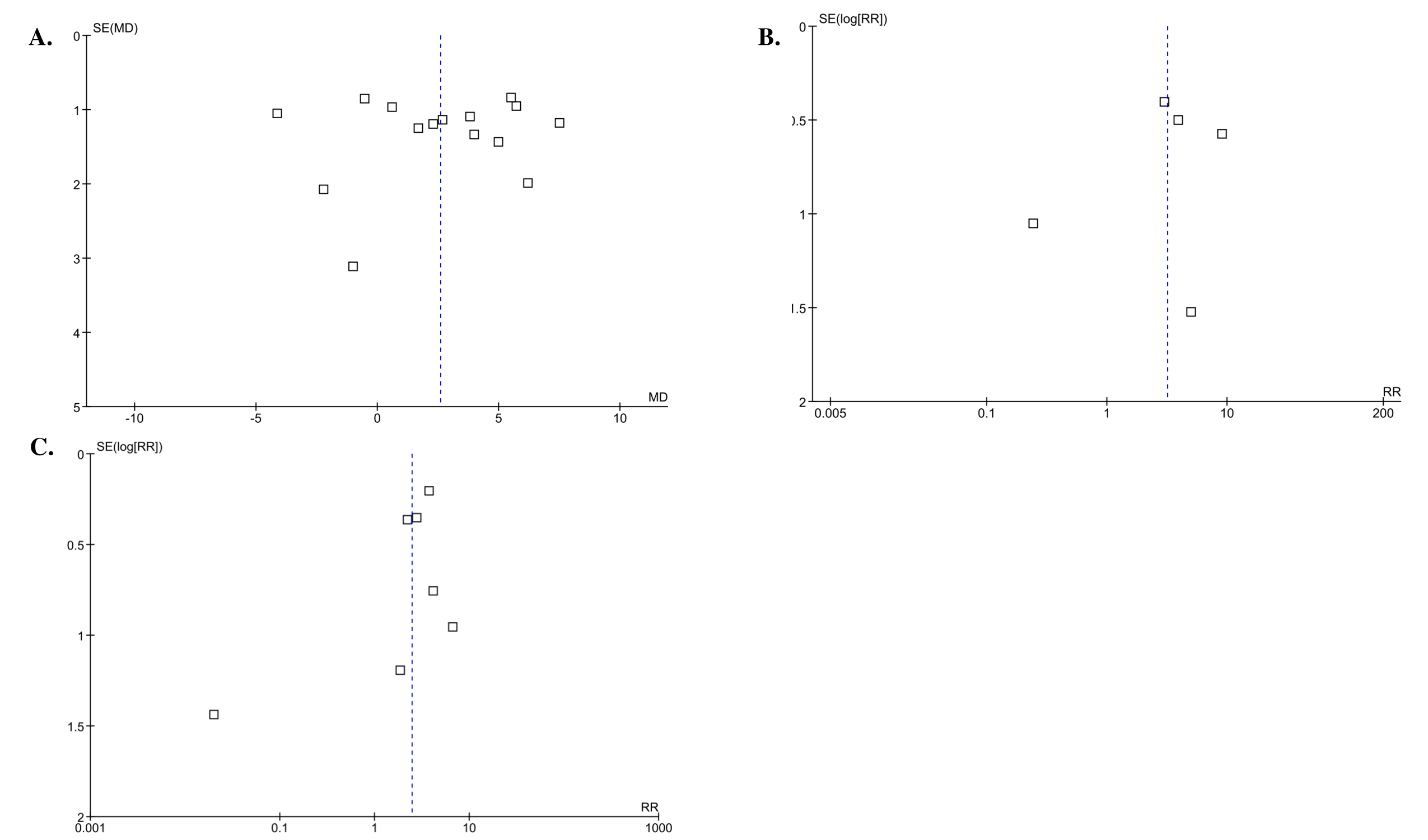

Supplement: Supplemental Digital Content [file jcarm-26-153-s006.tiff]

ROBINS-I Risk of Bias

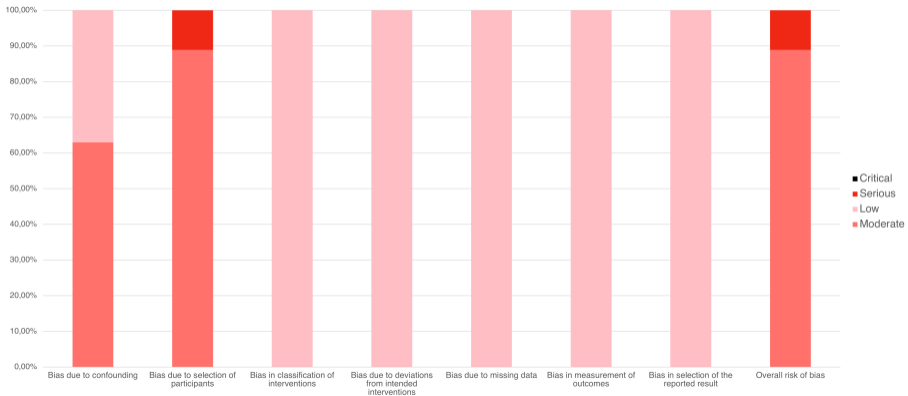

Supplement: Supplemental Digital Content [file jcarm-26-153-s007.pdf]
